# *KDM5C* and *KDM5D* mutations have different consequences in clear cell renal cell carcinoma cells

**DOI:** 10.1038/s42003-025-07695-8

**Published:** 2025-02-15

**Authors:** Marvin Müller, Kyra Zodel, Behnaz A. Abhari, Francesca Cuomo, Sheikh Nizamuddin, Patrick Metzger, Melanie Boerries, H. T. Marc Timmers, Ian J. Frew

**Affiliations:** 1 Department of Internal Medicine I, Hematology, Oncology and Stem Cell Transplantation, Faculty of Medicine, Medical Center—University of Freiburg, Freiburg, Germany; 2 Department of Urology, Faculty of Medicine, Medical Center—University of Freiburg, Freiburg, Germany; 3 Institute of Medical Bioinformatics and Systems Medicine, Faculty of Medicine, Medical Center—University of Freiburg, Freiburg, Germany; 4 Comprehensive Cancer Center Freiburg (CCCF), Faculty of Medicine, Medical Center—University of Freiburg, Freiburg, Germany; 5 German Cancer Consortium (DKTK), Partner Site Freiburg, Partnership Between the DKFZ and Medical Center—University of Freiburg, Freiburg, Germany; 6 Signalling Research Centre BIOSS, University of Freiburg, Freiburg, Germany

**Keywords:** Renal cell carcinoma, Epigenetics

## Abstract

*KDM5C* is commonly mutated in clear cell renal cell carcinomas (ccRCC) in men but rarely in women. Introducing *KDM5C* mutation into two male and two female *KDM5C* wild-type ccRCC cell lines caused different phenotypes and non-overlapping transcriptional consequences, indicative of context-dependent functions of KDM5C. We identify that loss of the Y chromosome, harbouring the *KDM5C* homologue *KDM5D*, occurs in most male *KDM5C* mutant ccRCCs. Mutation of *KDM5D* in male 786-O cells prevented xenograft tumour formation and this phenotype was unexpectedly rescued by co-mutation of *KDM5C*, consistent with the co-occurrence of *KDM5C* mutation and loss of the Y chromosome in ccRCC. Transcriptional analyses showed that KDM5C and KDM5D regulate the expression of both overlapping as well as distinct sets of genes. While KDM5C and KDM5D bind to at least some overlapping genomic sites, gene expression changes induced by *KDM5C* or *KDM5D* mutation are apparently unrelated to the direct functions of these proteins at the relevant gene promoters or enhancers. Our findings identify similarities and differences in KDM5C and KDM5D functions, challenging the idea that *KDM5D* in male cells functions equivalently to the second *KDM5C* allele in female cells, and implicate an interplay between *KDM5C* mutation and Y chromosome loss in ccRCC development in men.

## Abstract (web summary)

This study challenges the idea that KDM5D in male cells functions equivalently to the second KDM5C allele in female cells and implicates an interplay between KDM5C mutation and Y chromosome loss in ccRCC development in men.

## Introduction

Clear cell renal cell carcinoma (ccRCC) represents 70–80% of all renal malignancies and is one of the ten most frequently occurring tumours in adults. ccRCC exhibits a unique mutation spectrum which suggests that it is an epigenetically-driven tumour. Eighty-two to ninety-two per cent of ccRCC primary tumours harbour biallelic inactivation of the von Hippel–Lindau (*VHL*) tumour suppressor gene due to loss of one copy of chromosome 3p and inactivation of the second allele by mutation, deletion or hypermethylation^1,2^. Biallelic *VHL* inactivation is known to be the first event in the process of tumour formation in the majority of ccRCC cases^1–5^. The resulting activation of HIF-1α and HIF-2α transcription factors causes the transcriptional upregulation of several histone demethylases including *KDM3A*, *KDM4B*, *KDM5C* and *KDM6B*^6–10^, implicating various alterations in the methylation of histone H3 as an early event in the process of tumour formation following *VHL* inactivation. Indeed, human ccRCC tumours were shown to have a distinctive histone modification profile, being characterised amongst other modifications by decreased cellular levels of tri-methylation of lysine-27 of histone H3 (H3K27me3) and increased levels of acetylation of lysine-27 of histone H3 (H3K27ac)^11^, a histone modification that is typically associated with active gene transcription. Further investigations revealed that loss of *VHL* function caused accumulation of active chromatin marks including H3K27ac and mono-methylation of lysine-4 of histone H3 (H3K4me1) specifically at gene enhancers, due at least in part to the binding of stabilised HIF-2α to these regions and the recruitment of the p300 histone acetylase^12^. Thus, as the earliest event in tumour formation, *VHL* mutation appears to induce alterations in cellular epigenetics via a number of HIF-α-dependent mechanisms. During ccRCC formation, cells accumulate additional mutations in other epigenetic regulatory genes^1–5^. These mutations include *PBRM1*, encoding a crucial subunit of the PBAF SWI/SNF chromatin remodelling complex, *SETD2* encoding the histone H3 lysine-36 (H3K36) trimethylase, *BAP1* encoding the histone H2A lysine-119 (H2AK119) deubiquitinase and *KDM5C* encoding a histone H3-lysine 4 (H3K4) di-/tri-methyl demethylase. *KDM5C* mutations were observed in 7% of single biopsies sampled from primary ccRCC^1^. However, sampling from multiple regions identified intra-tumoural clones with *KDM5C* mutations in 20% of primary ccRCCs^4^, a mutational frequency similar to the 18.4%^13^ and 24%^14^ mutational frequencies observed in two studies of ccRCC metastases. These genetic observations collectively argue that alterations in specific aspects of normal epigenetic regulation through post-translational regulation of histones are likely to be particularly important in the process of malignant transformation of normal renal epithelial cells and in the progression of ccRCC tumours.

Men are approximately twice as likely as women to develop ccRCC. The molecular basis of the sex-specific difference in ccRCC is unknown. In this context, the X chromosome encoded *KDM5C* is a particularly interesting gene as it is one of the few genes that are not subject to X-inactivation in females, meaning that both *KDM5C* alleles are expressed in female cells^15^. The Y chromosome harbours the *KDM5D* gene, encoding the KDM5D protein, which exhibits 86% amino acid identity and 91% amino acid similarity to KDM5C. KDM5C and KDM5D share the biochemical function of acting as specific demethylases for di- and tri-methylated H3K4, converting H3K4 to the mono-methylated form in vitro assays^16^. Nucleosomes marked by H3K4me3 recruit transcriptional activators like TFIID and SAGA, but repressors like SIN3A can also bind H3K4me3. The major H3K4 methylases in humans are the six SET1/MLL complexes, of which the genes encoding the MLL3 and MLL4 subunits are frequently mutated in cancer. Their activity is balanced by four KDM5 and two KDM1 demethylases. While nothing is known about whether they have similar or different functions in the kidney, it is believed that in female cells, two expressed alleles of *KDM5C* are required for normal cellular functions, whereas in male cells, Y chromosome-encoded *KDM5D* serves as the surrogate second allele to complement the single X chromosome encoded *KDM5C* allele. It is therefore intriguing that *KDM5C* is more frequently mutated across all types of cancers, including in ccRCC, in males than in females^17^. In ccRCC this gender bias in gene mutation frequency is noteworthy because 30–40% of male ccRCCs demonstrate loss of the Y chromosome, which deletes amongst others the *KDM5D* gene^17,18^. Analyses of exome sequencing data from 41 single biopsies of primary ccRCC tumours in females revealed that 3 tumours harboured *KDM5C* mutations and showed concomitant loss of the X chromosome but an additional 6 tumours that harboured deletions of *KDM5C* due to loss of part or all of the X chromosome did not show mutations in *KDM5C*^18^. KDM5C inactivation can therefore be heterozygous or nullizygous in female ccRCC. Of 52 primary ccRCC tumours in males, 19 showed loss of the Y chromosome and of these, only 1 showed mutation of *KDM5C* whereas 2 tumours with an intact Y chromosome showed *KDM5C* mutation^18^. Our own unpublished exome sequencing of metastatic ccRCC samples from 9 male patients revealed that 6 tumours exhibited loss of the Y chromosome, 2 of these tumours harboured *KDM5C* mutations and 1 additional tumour with an intact Y chromosome showed a *KDM5C* mutation. It appears that male ccRCCs may be deficient in either *KDM5C* or *KDM5D* or both. Taken together, any one of either *KDM5C* mutation, loss of part or all of the X chromosome or loss of part or all of the Y chromosome causing loss of *KDM5D* might act to promote tumour formation, (i.e. the genes function as haploinsufficient tumour suppressors), but that rare bi-allelic inactivation of *KDM5C* in females or combined mutation of *KDM5C* and loss of the Y chromosome in males might also be selected for in some tumours, potentially providing additional attributes to those tumours. We aimed to test these ideas in this study by studying the effects of mutation of *KDM5C* and/or *KDM5D* in human ccRCC cell lines.

## Results

### *KDM5C* mutation has different effects on cancer cell behaviour in different ccRCC cell lines

**Fig. 1:**
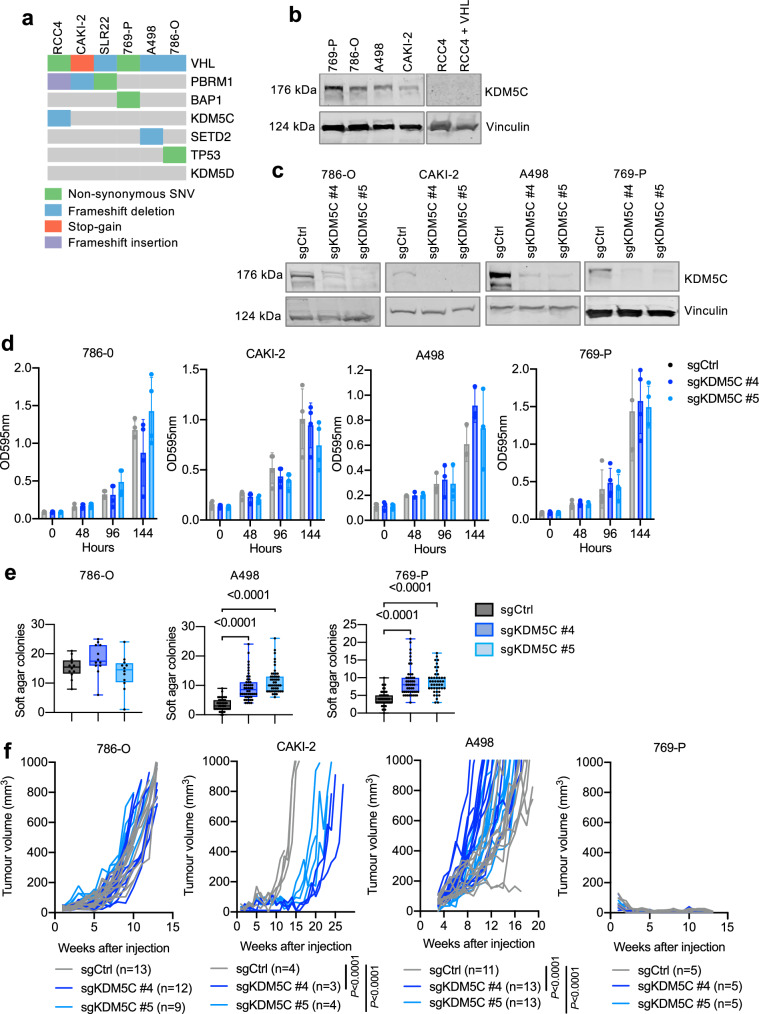
Different effects of *KDM5C* mutation in different ccRCC cell lines. **a** Oncoprint showing gene mutations in human ccRCC cell lines. **b** KDM5C western blot of ccRCC cell lines. **c** KDM5C western blot in the indicated cell lines infected with lentiviruses expressing sgCtrl, sgKDM5C #4 or sgKDM5C #5 together with spCas9. Predicted molecular masses are shown. Full western blot scans including molecular mass standards are provided in Supplementary Fig. 6. **d** Crystal violet assay (OD595nm) of the time course of the proliferation of the indicated cell lines (*n* = 3–4, mean ± std. dev.). **e** Soft agar colony formation assay of the indicated cell lines (*n* = 12–48, mean ± std. dev.). Statistical differences were calculated using one-way ANOVA with Dunnett’s multiple comparisons test. **f** Xenograft assays of the indicated cell lines showing the growth of each individual tumour (*n* values represent numbers of independent tumours per condition). Statistical differences in the sizes of tumours in each group were calculated using one-way ANOVA with Dunnett’s multiple comparisons test.

Across all human cancer types, *KDM5C* mutations arise most frequently in endometrial, renal and lung tumours (Supplementary Fig. 1a). In ccRCC, *KDM5C* mutations are found at increased frequency in sub-clones within primary tumours as well as in metastases, in comparison to single biopsies of primary tumours (Supplementary Fig. 1b), indicating that they arise later during the process of ccRCC evolution rather than being truncal mutations. Consistent with this observation, *KDM5C* mutations in ccRCC metastasis frequently arise together with different combinations of other recurrently altered ccRCC tumour suppressor genes including *VHL*, *PBRM1*, *SETD2*, *TP53*, *PTEN* or with the *MTOR* oncogene (Supplementary Fig. 1c). Mutations in *KDM5C* that occur across all tumour types, including in ccRCC, papillary RCC and chromophobe RCC tumours show the typical pattern of inactivation of a tumour suppressor gene, being present throughout the coding region (Supplementary Fig. 1d). Across all tumour types 22% of these mutations are truncating, which are predicted to completely disrupt the function of the protein product. Interestingly, in RCC tumours, 67% of *KDM5C* mutations are truncating, suggestive of a strong selective pressure to completely inactivate KDM5C function in this tumour type. To mimic the effects of loss of function mutations in *KDM5C* in the background of other ccRCC-specific mutations we employed CRISPR-Cas9 mutagenesis to compare the effects of *KDM5C* mutation across multiple ccRCC cell lines. Exome-sequencing^19^ (Fig. 1a) identified that the 786-O, CAKI-2 and SLR22 male human ccRCC cell lines show no *KDM5C* or *KDM5D* mutations and that the female A498 and 769-P cell lines also have wild type *KDM5C*. RCC4 cells exhibit a *KDM5C* frameshift deletion as previously described^20^. *KDM5C* mRNA was detected at similar levels in 786-O, CAKI-2, A498 and 769-P cells (Supplementary Fig. 2) and western blotting confirmed the expression of KDM5C in all four cell lines with *KDM5C* mutant RCC4 cells serving as a negative control for the antibody (Fig. 1b). Variations in KDM5C protein abundance relative to the Vinculin loading control did not correlate with sex of the cell line, with male 786-O and female A498 cells expressing similar levels, male CAKI-2 cells expressing lower levels and female 769-P cells expressing the highest levels. We designed two independent sgRNAs (#4 and #5) targeting exon 8 of *KDM5C*, designed to induce mutations in the first PHD domain, and infected populations of cells with MuLE lentiviruses expressing Cas9, puromycin resistance and the individual sgRNAs or a non-targeting control (sgCtrl)^21^. Puromycin-selected stably transduced cells with sg*KDM5C* #4 and sg*KDM5C* #5 showed a strong reduction of KDM5C protein abundance in all cell lines (Fig. 1c). Thus, we successfully generated populations of 786-O, CAKI-2, A498 and 769-P cells harbouring different Cas9-induced *KDM5C* mutations that cause loss of protein abundance, avoiding the potentially confounding effects of clonal artefacts that can arise from knockout approaches in which single cells are selected. *KDM5C* mutation did not affect the proliferation of any of the cell lines (Fig. 1d). *KDM5C* mutant A498 and 769-P cells formed more colonies in soft-agar assays than control cells, while *KDM5C* mutation did not affect 786-O cell anchorage-independent growth in soft agar (Fig. 1e). Neither control nor *KDM5C* mutant CAKI-2 cells formed colonies in this assay. *KDM5C* mutation did not alter xenograft tumour formation by 786-O cells, slowed xenograft growth in CAKI-2 cells, increased xenograft growth in A498 cells and did not allow xenograft tumour formation in 769-P cells, a cell line in which control cells do not form tumours (Fig. 1f). Thus, *KDM5C* mutation has cell line-specific consequences on several aspects of transformed behaviour of ccRCC cancer cells.

### *KDM5C* mutation causes non-overlapping transcriptional changes in different ccRCC cell lines

**Fig. 2:**
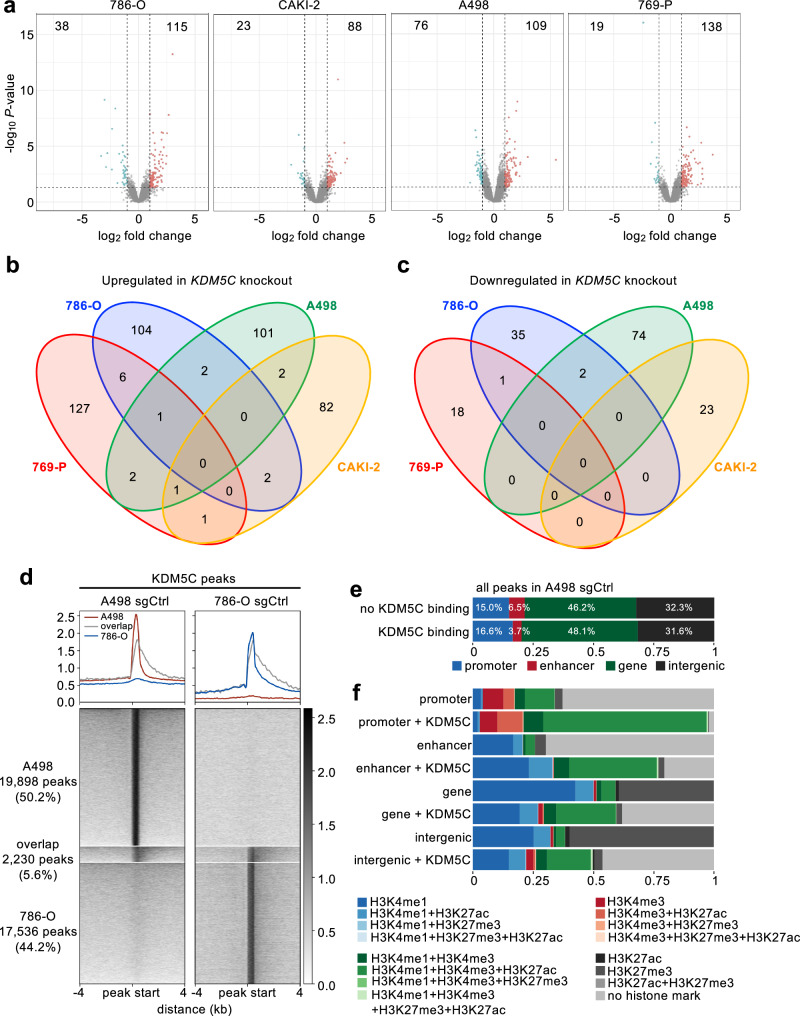
*KDM5C* mutation induces non-overlapping gene expression changes in different ccRCC cell lines. **a** Volcano plot showing differentially expressed genes (*p* < 0.05, log_2_fold change < −1 or >1) comparing the two sgKDM5C to sgCtrl cells for each cell line. Blue colour highlights significantly downregulated genes, red colour highlights significantly upregulated genes. Corresponding numbers display the number of upregulated and downregulated genes, respectively. **b**, **c** Venn diagrams showing overlapping upregulated (**b**) and downregulated (**c**) genes in *KDM5C* mutant cells across the four cell lines. **d** CUT&RUN KDM5C binding sites in A498 and 786-O cells displayed as profiles (upper panels) and heatmaps (lower panels). **e** Percentage of genomic features with or without KDM5C binding in A498 control cells. **f** Distribution of histone mark patterns (colours) at genomic features with or without detected KDM5C binding (rows).

Activation of gene transcription is strongly associated with methylation of lysine-4 of histone H3 (H3K4). Mono-methylation of this residue (H3K4me1) is enriched at transcriptional enhancers, while tri-methylation (H3K4me3) is a strong predictor of active gene promoters. In mouse ES cells KDM5C is recruited to both promoters and enhancers and normally acts to repress promoters by reducing H3K4me3 levels and acts to promote enhancer function by maintaining the H3K4me1 state^22^. To test whether genetic mutations of *KDM5C* similarly affect gene expression in ccRCC cells we conducted RNA-seq of our panel of cell lines. In each cell line, *KDM5C* mutant cells exhibited only modest transcriptional changes compared to sgCtrl cells (Fig. 2a) and there was no overlap in the differentially regulated genes between all of the 786-O, CAKI-2, A498 and 769-P cell lines (Fig. 2b). Only one or two genes were commonly up- or down-regulated between any two cell lines (Fig. 2b). This finding of modest and cell line-specific effects of *KDM5C* mutation on gene transcription is consistent with the fact that large-scale genomic studies such as TCGA did not identify any specific transcriptional profiles associated with *KDM5C* mutant ccRCCs.

Comparison of gene set enrichment analyses across the different cell lines did not identify consistent similarities or differences in biological pathways that could explain the differential effects of *KDM5C* mutation on xenograft tumour growth (Supplementary Fig. 3a). For example, *KDM5C* mutant A498, CAKI-2 and 786-O cells all showed enrichment of the predicted pro-proliferative Hallmark terms E2F targets and Myc targets, yet *KDM5C* mutation enhanced, inhibited or had no effect on xenograft growth of these cells, respectively. In CAKI-2 cells, the enrichment of the GO term “Negative regulation of cell cycle processes” and downregulation of genes associated with GO, Hallmark and KEGG terms “Oxidative phosphorylation” are potentially consistent with the delay in xenograft growth induced by *KDM5C* mutation in this cell line.

### KDM5C binding does not correlate with transcriptional changes

We next used CUT&RUN to identify genome-wide binding profiles of KDM5C in female A498 and male 786-O ccRCC control cells. Only 5.6% of KDM5C binding sites overlapped between the two cell lines (Fig. 2d), which is in accordance with the cell line-specific transcriptomic effects upon loss of *KDM5C*.

To investigate whether KDM5C affects gene transcription by directly regulating gene promoters or enhancers we focused further analyses on A498 cells as this cell line is female, avoiding potential complications of KDM5D function, and since we showed that KDM5C functions as a tumour suppressor in A498 cells in the context of the xenograft assay. KDM5C binding sites were further characterized by identifying co-localised CUT&RUN peaks for H3K4me1, H3K4me3, H3K27me3, and H3K27ac and by determining overlaps with genomic features such as promoters and enhancers. Due to the complexity of assigning target genes to their respective enhancers, only enhancer coordinates of known enhancer-gene pairs could be used, which does not represent a complete list of putative enhancers.

Considering all KDM5C peaks detected in A498 control cells, the majority were located within gene coordinates, especially introns, and intergenic regions (48.1% and 31.6%, respectively). 16.6% of KDM5C was bound within promoters and 3.7% within enhancers (Fig. 2e). The intersection of all histone mark binding sites revealed multiple combinations of overlapping histone marks, which were not equally distributed across the genomic features (Fig. 2f). This observation is consistent with the fact that histone mark patterns can be indicative for specific transcriptional states and can feature regulatory elements such as primed or active promoters and active enhancers. Active promoters are characterized by high levels of H3K4me3 and H3K27ac and active enhancers show high levels of H3K4me1 and H3K27ac without H3K27me3. Interestingly, promoters, enhancers, genes and intergenic regions to which KDM5C was bound were enriched for the combination of H3K4me1, H3K4me3 and H3K27me3 compared to annotated but non-KDM5C bound promoters, enhancers, genes and intergenic regions (Fig. 2f), which is consistent with the known biochemical function of KDM5C in converting H3K4me3 into H3K4me1. Genes and intergenic regions that were bound by KDM5C were characterised by the almost complete absence of H3K27me3 and H3K27ac compared to annotated regions that did not show KDM5C peaks (Fig. 2f). We conclude that KDM5C binding is associated with different patterns of histone modifications at promoters, enhancers, genes and intergenic regions of the genome.

**Fig. 3:**
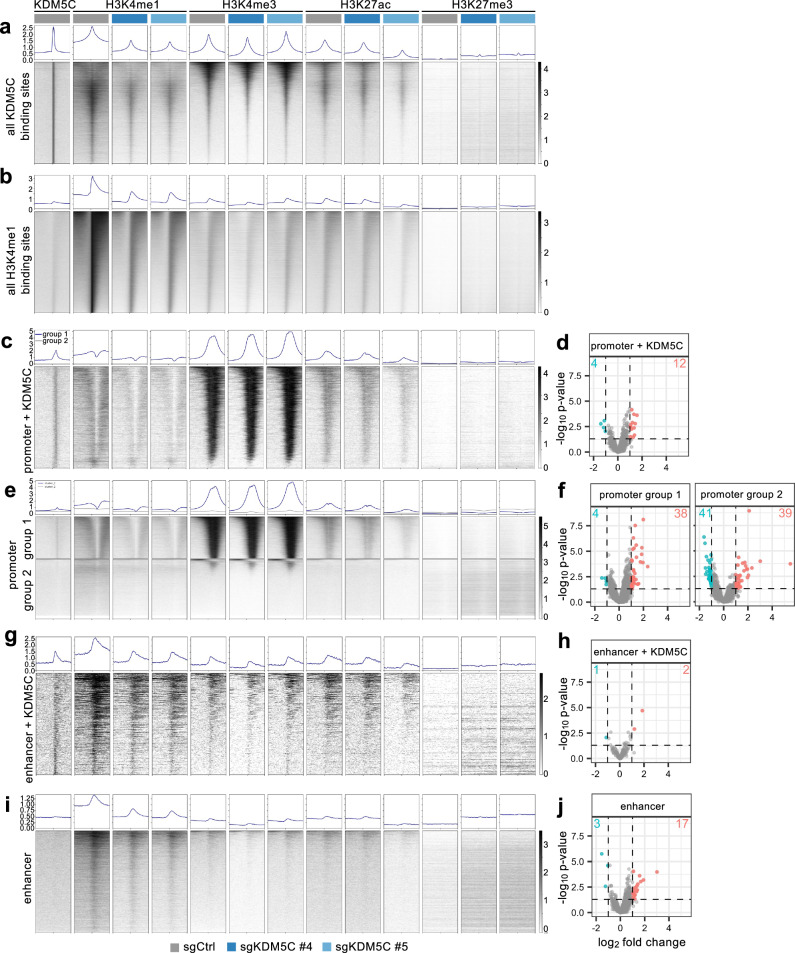
KDM5C binding at promoters and enhancers and associated gene expression changes in A498. **a**–**c**, **e**, **g**, **i** Heatmaps and profiles of CUT&RUN signals for KDM5C and the indicated histone H3 marks in sgCtrl, sgKDM5C #4 and sgKDM5C #5 A498 cells 4 kb upstream and downstream for the following sets of sites: (**a**) 22,195 identified KDM5C binding sites, (**b**) 102,500 identified H3K4me1 sites, (**c**) 2444 promoter coordinates for 2385 unique genes with KDM5C binding, (**e**) promoter elements lacking KDM5C binding (group 1 shows 14,759 peaks for 10,636 unique genes, group 2 shows 20,978 peaks for 16,333 unique genes), (**g**) 651 kidney enhancer coordinates for 638 unique genes with KDM5C binding, (**i**) 13,394 kidney enhancer coordinates for 10,664 unique genes without KDM5C binding. **d**, **f**, **h**, **j** Volcano plot showing differentially expressed genes (*p* < 0.05, log_2_fold change < −1 or >1) comparing the two sgKDM5C to sgCtrl A498 cells for each set of genes associated with the CUT&RUN categories. **d** One thousand nine hundred fifty-four genes corresponding to promoters with KDM5C binding, (**f**) 8435 genes (group 1) and 2546 genes (group 2) corresponding to promoters without KDM5C binding, (**h**) 202 genes corresponding to enhancers with KDM5C binding, (**j**) 1831 genes corresponding to enhancers without KDM5C binding.

In order to assess the correlation of KDM5C binding with gene expression, KDM5C binding sites and histone modifications were explored in more detail in sgCtrl and sgKDM5C #4 and sgKDM5C #5 A498 cells. Heatmaps revealed that across all KDM5C binding sites, levels of H3K4me1 were reduced in the *KDM5C* mutant cells, consistent with KDM5C functioning as a major H3K4 demethylase that converts di- and tri-methylated H3K4 into the mono-methylated form (Fig. 3a). In the absence of KDM5C, these sites can no longer be converted to the H3K4me1 form. KDM5C also colocalized with regions of both high and low H3K4me3 signals (Fig. 3a). A fraction of the regions with high H3K4me3 signal showed low levels of H3K4me1 and increased levels of H3K27ac which could be indicative of active promoters. We also noted that when aligning all H3K4me1 peaks in wild-type cells, the same peaks were less intense in the two *KDM5C* mutant cell lines (Fig. 3b), indicating that *KDM5C* deficiency globally reduces the abundance of the histone mark that is expected to be formed by KDM5C activity. This observation suggests that the KDM5C CUT&RUN may not identify all sites of KDM5C binding or that there are sites of stable H3K4me1 that do not require ongoing KDM5C binding to maintain them in the monomethylated state.

We hypothesised that KDM5C activity at active promoters might normally inhibit gene transcription by demethylating the active promoter mark H3K4me3 and hence loss of KDM5C activity at these sites might cause upregulation of some of these genes in the *KDM5C* mutant cells. To test this idea, all RefSeq hg19 promoter coordinates were grouped into promoters with or without detected KDM5C binding and the histone mark signals were visualised by heatmaps. For promoters with detected KDM5C binding, the H3K4me3 and H3K27ac levels at these sites were high, while the H3K4me1 signal was mostly low (Fig. 3c). Surprisingly, of the genes associated with these promoters only 12 were upregulated and 4 downregulated in *KDM5C* mutant cells (Fig. 3d). In contrast, histone mark patterns of promoters without detected KDM5C binding could be divided into two major groups (Fig. 3e), the first being characterized by high H3K4me3 and low H3K4me1 signals with moderate levels of H3K27ac. Promoters in this group were associated with 38 of the total 109 upregulated genes and 4 of the total 76 downregulated genes in *KDM5C* mutant cells (Fig. 3f). The second promoter group showed no signal for any histone mark but 39 of the genes associated with these promoters were upregulated and 41 were downregulated in *KDM5C* mutant cells (Fig. 3f). Thus, 70% of the genes that are upregulated in *KDM5C* knockout cells and 59% of the downregulated genes were associated with promoters to which KDM5C binding was not detected. Only 11% of the upregulated genes and 5% of the downregulated genes were associated with KDM5C-bound promoters. These observations suggest that the predominant mode of KDM5C-mediated control of gene expression is not via direct binding and regulation of H3K4 methylation at promoters. It is also possible that the majority of the observed gene expression changes are due to secondary effects of *KDM5C* mutation rather than being direct target genes.

We also investigated the idea that KDM5C could activate gene transcription via enhancers by maintaining the active enhancer mark H3K4me1. Enhancers are mostly distal regulatory elements and the assignment of target genes remains challenging. Additionally, enhancers are very cell-type-specific and depend on developmental states. The GeneHancer database comprises genome-wide enhancers supported by different sources and enhancer-gene associations are scored based on multiple methods. All available enhancer coordinates from GeneHancer kidney tissues were similarly grouped into enhancers with or without KDM5C binding. Enhancers with KDM5C binding showed enrichment of H3K4me1, H3K4me3, and H3K27ac (Fig. 3g) and of these marks, only H3K4me1 levels were decreased in *KDM5C* mutant cells (Fig. 3g), consistent with the anticipated function of KDM5C at enhancer elements. However, only two genes and one gene associated with these enhancers were up- or down-regulated in *KDM5C* mutant cells, respectively (Fig. 3h). Enhancers without KDM5C binding showed some H3K4me1 signals which were also reduced in *KDM5C* mutant cells (Fig. 3i). 17 and 3 genes associated with these enhancers were up- or down-regulated in *KDM5C* mutant cells, respectively (Fig. 3j). These analyses show that the genes that are differentially expressed in *KDM5C* mutant cells are in fact mostly associated with promoter or enhancer sites that are not bound by KDM5C. This conclusion is supported by the reverse visualisation in which the promoter and enhancer regions of only the up- and down-regulated genes are shown as heatmaps depicting the patterns of binding of KDM5C and peaks for H3K4me1, H3K4me3, H3K27ac and H3K27me3 (Supplementary Fig. 3b–e). No enrichment of KDM5C binding at these sites is observed, however reduction of H3K4me1 is seen at all of these sites in *KDM5C* mutant cells, potentially consistent with a global effect of loss of KDM5C function on H3K1me1 levels.

### KDM5C and KDM5D have different functions in 786-O ccRCC cells

**Fig. 4:**
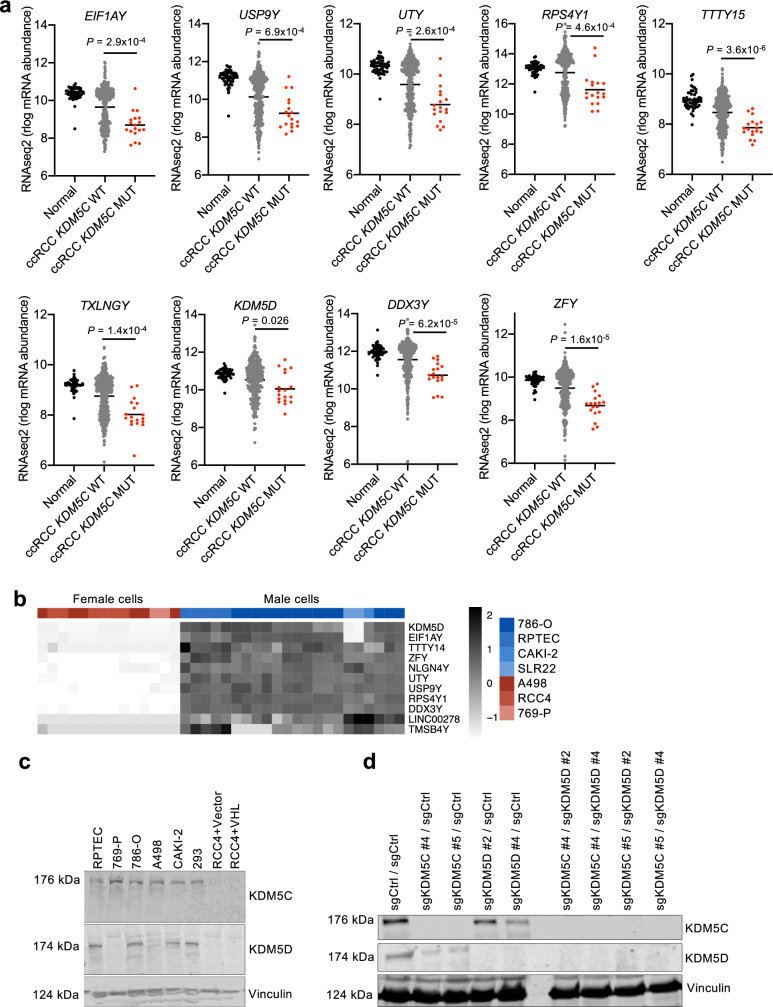
Y chromosome loss in *KDM5C* mutant human ccRCC tumours. **a** Expression levels of Y chromosome genes in normal male kidney and in *KDM5C* wild type or mutant male ccRCC, RNAseq2 quantification from the KIRC TCGA study. Statistical differences were assessed using a two-sided Student’s unpaired *t*-test with Welch’s correction. **b** mRNA expression levels (*z*-scores) of Y chromosome genes in human ccRCC cell lines. **c** KDM5C and KDM5D western blots in the indicated cell lines. **d** KDM5C and KDM5D western blots in the 786-O cells infected with lentiviruses expressing the indicated combinations of sgCtrl, sgKDM5C #4, sgKDM5C #5, sgKDM5D #2 and sgKDM5D #4 together with spCas9. Predicted molecular masses are shown. Full western blot scans including molecular mass standards are provided in Supplementary Fig. 6.

Previous studies revealed that the vast majority of *KDM5C* mutations are found in male ccRCCs and that the Y chromosome, harbouring *KDM5D*, is lost in approximately 40% of male ccRCC tumours^17,18^. The relationship between *KDM5C* mutation and Y chromosome loss however has not been investigated in large enough numbers of samples to provide more than anecdotal observations. While standard bioinformatic pipelines to call copy number variations from Exome-seq data, such as those used by the TCGA studies, do not assess the status of the Y chromosome, we took advantage of TCGA KIRC mutation and RNA-seq data to analyse the expression levels of nine Y chromosome-encoded genes, including *KDM5D*, as a surrogate readout of loss of the Y chromosome. A similar approach was recently employed to assess Y chromosome loss in bladder cancer^23^. Male ccRCC tumours harbouring a *KDM5C* mutation showed significantly lower levels of expression of all nine Y chromosome genes than *KDM5C* wild-type tumours (Fig. 4a). Tumours segregated into a group with gene expression levels approximately equivalent to those in normal tissue and a group with low levels of expression. The group with low Y chromosome gene expression in *KDM5C* wild-type tumours represented approximately 40% of the cases, similar to the previously reported frequency of loss of the Y chromosome in ccRCC^17,18^ while depending on the gene, between 12 and 16 of the 17 total (71–94%) *KDM5C* mutant tumours showed low Y chromosome gene expression. Thus, *KDM5C* mutation in male tumours is very frequently associated with low levels of Y chromosome gene expression, which very likely indicates loss of the Y chromosome.

**Fig. 5:**
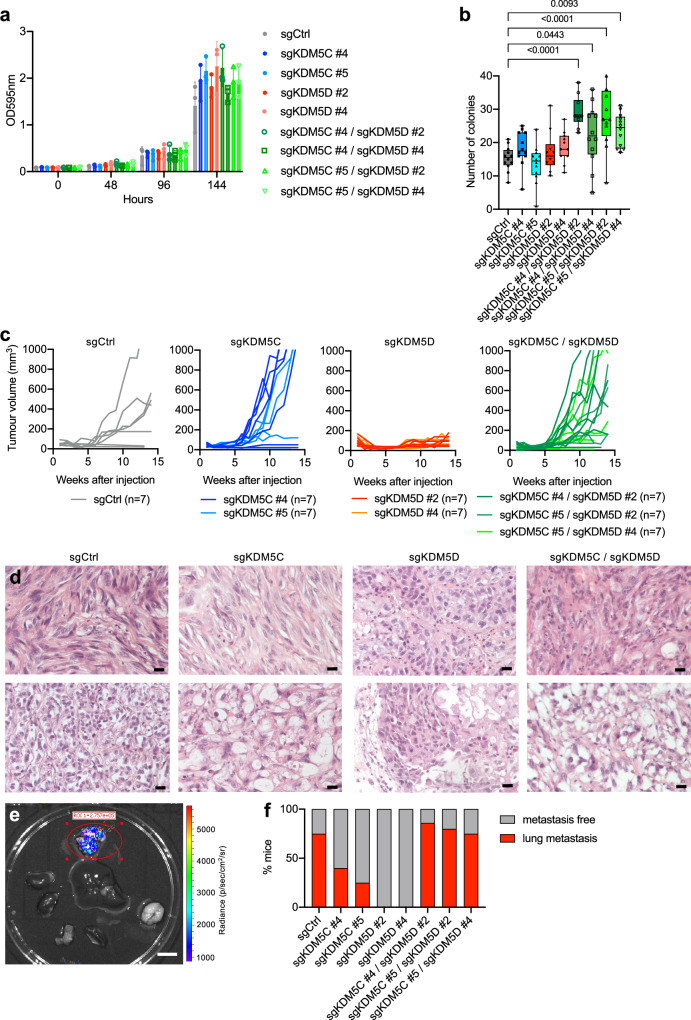
Effects of *KDM5C* and *KDM5D* mutations on cancer phenotypes of 786-O cells. **a** Crystal violet assay (OD595nm) of the time course of the proliferation of the indicated cell lines (*n* = 3, mean ± std. dev.). **b** Soft agar colony formation assay of the indicated cell lines (*n* = 10–12, mean ± std. dev.). Statistical differences were calculated using one-way ANOVA with Dunnett’s multiple comparisons test. **c** Xenograft assays of the indicated cell lines showing the growth of each individual tumour. **d** Representative images of the range of histological appearances of tumours of the indicated genotypes. Scale bars = 20 μm. **e** Representative luciferase image showing lung metastasis. Scale bar = 11 cm. **f** Frequency of lung metastases of the indicated genotypes.

We next looked for an appropriate ccRCC cell line to allow investigation of the effects of *KDM5C* and *KDM5D* mutation in ccRCC. We analysed expression levels of Y chromosome encoded genes in the 786-O, CAKI-2 and SLR22 male ccRCC cell lines, using normal male renal proximal tubule epithelial cells (RPTEC) as a positive control and female ccRCC cell lines as negative control (Fig. 4b). 786-O and CAKI-2 cells robustly express Y chromosome genes, while SLR22 cells express *KDM5D* and *EIF1AY* at very low levels, suggestive of a partial deletion of the Y chromosome, so were not considered for further genetic knockout experiments. Of note, *KDM5C* mRNA levels are 4–7 times higher than *KDM5D* mRNA levels in RPTEC, 786-O and CAKI-2 cell lines (Supplementary Fig. 3). Western blotting confirmed the expression of both KDM5C and KDM5D in 786-O and CAKI-2 cells (Fig. 4c). Since 786-O cells are also wild type for the other major ccRCC epigenetic tumour suppressor genes *PBRM1*, *BAP1* and *SETD2* (Fig. 1a), we chose these cells for further study. We used Cas9-expressing MuLE lentivirus to generate populations of cells with mutations in exon 2 of *KDM5D* (sgKDM5D #2) or exon 4 of *KDM5D* (sgKDM5D #4) (Fig. 4d). We also generated four different populations of 786-O cells expressing the different combinations of the two independent *KDM5C* sgRNAs and the two independent *KDM5D* sgRNAs (Fig. 4d). All single and double mutant cell populations proliferated equivalently in normal cell culture conditions (Fig. 5a) but the double knockout cells formed more colonies in soft-agar (Fig. 5b). This finding is notable as it mimics the effect of *KDM5C* mutation in the two female ccRCC cells (Fig. 1e), suggestive of overlapping functions of KDM5C and KDM5D and mutual compensation for the loss of one of the proteins. Consistent with the previous experiment (Fig. 1f), *KDM5C* mutation did not affect xenograft tumour growth (Fig. 5c). Strikingly however, *KDM5D* mutant cells failed to form xenograft tumours but the combination of *KDM5C* and *KDM5D* mutations restored the ability to form tumours (Fig. 5c). All sgCtrl, sgKDM5C and sgKDM5C/sgKDM5D tumours displayed mixed histological appearances with regions of clear cell or eosinophilic squamous-like growth pattern intermixed with regions of clear cell morphology organised in an acinar pattern. Consistent with the failure to form tumours, the small nests of remnant injected sgKDM5D cells that could be detected in a few injected animals showed a less transformed appearance of large clear cells with larger nuclei growing in an acinar pattern (Fig. 5d). Mutation of *KDM5C* therefore restores the non-transformed appearance of *KDM5D* mutant cells. Taking advantage of the fact that the cells injected for xenograft assays were labelled with luciferase (Fig. 5e) we noted that *KDM5C* mutant tumours showed a non-significant trend to metastasise less frequently to the lungs than control 786-O cells and co-mutation of *KDM5D* in *KDM5C* mutant tumours restored the frequency of lung metastatic spread to a similar level to sgCtrl cells (Fig. 5f). In summary, *KDM5C* mutation allows *KDM5D* mutant cells to form tumours and *KDM5D* mutation appears to restore metastatic capacity of *KDM5C* mutant cells. This genetic relationship between *KDM5C* and *KDM5D* mutation is consistent with the human data that indicates that *KDM5C* mutant male ccRCC tumours display concomitant loss of *KDM5D* function through Y chromosome loss.

### KDM5C and KDM5D differently affect gene expression in 786-O ccRCC cells

**Fig. 6:**
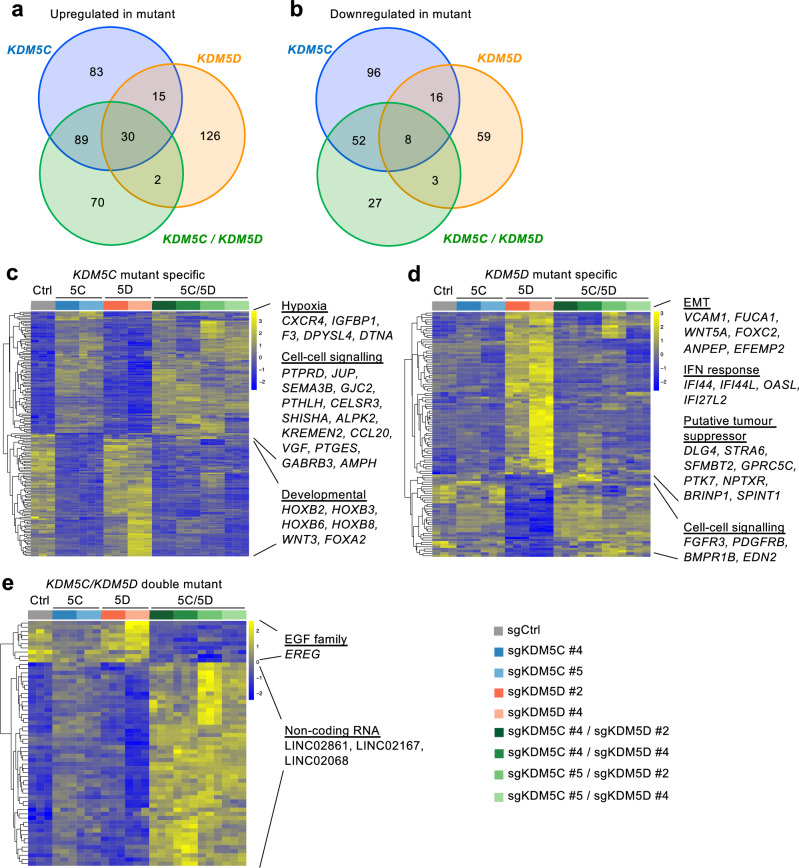
Different and antagonistic effects of *KDM5C* and *KDM5D* mutation on gene expression in 786-O cells. **a**, **b** Venn diagrams showing overlapping upregulated (**a**) and downregulated (**b**) genes between *KDM5C, KDM5D* and double *KDM5C/KDM5D* mutant cells versus sgCtrl cells (fold change >1.5 or <-1.5, adjusted *p* < 0.001). **c–e** Heatmaps (*z*-scores) and selected genes that are specific to (**c**) *KDM5C* mutation (80 upregulated, 81 downregulation), (**d**) to *KDM5D* mutation (73 upregulated, 37 downregulated), or (**e**) to double *KDM5C/KDM5D* mutation (49 upregulated, 10 downregulated). **c**, **d** Genes were filtered based on an adjusted *p*-value < 0.001 and fold change > 1.5 or <−1.5 and any overlapping genes between KDM5C and KDM5D knockouts were removed. **e** Genes showing a stronger effect in *KDM5C/KDM5D* double mutant cells were filtered based on an adjusted *p*-value < 0.001 and fold change > 1.5 or <−1.5 in *KDM5C/KDM5D* double mutant cells versus control cells and for each gene the mean log_2_ fold change was >1.3-fold higher than in either of the single mutant cells.

To investigate whether transcriptional changes might underlie the different effects of *KDM5C* and *KDM5D* mutation on cancer cell behaviours, we conducted RNA-seq of triplicate samples of all 8 mutant 786-O cell populations and identified up- and down-regulated genes compared to sgCtrl cells (Supplementary Data File 3). *KDM5C* and *KDM5D* single mutations shared 45 upregulated genes but displayed 172 and 128 unique upregulated genes, respectively (Fig. 6a). Similarly, *KDM5C* and *KDM5D* single mutations shared only 24 downregulated genes but displayed 148 and 62 unique downregulated genes, respectively (Fig. 6b). The transcriptional consequences of *KDM5C* and *KDM5D* mutations are therefore partly overlapping but are predominantly distinct from one another. About half of the genes that were upregulated in *KDM5C/KDM5D* double mutant cells were also upregulated in *KDM5C* single mutant cells but only 2 were also upregulated in *KDM5D* mutant cells (Fig. 6a). Only 27 genes were downregulated uniquely in *KDM5C/KDM5D* double mutant cells and the other 63 genes were also downregulated in *KDM5C* or *KDM5D* mutant cells (Fig. 6b). Genes whose expression was up- or downregulated in *KDM5C* mutant but not in *KDM5D* mutant cells (Fig. 6c), up- or downregulated in *KDM5D* mutant but not in *KDM5*C mutant cells (Fig. 6d), or up- or downregulated only in *KDM5C/KDM5D* double mutant cells but not in either single mutant (Fig. 6e) are depicted as heatmaps. *KDM5C* mutation-specific up-regulated genes include hypoxia targets and genes involved in cell-cell signalling, while a series of developmental genes (*HOXB2, HOXB3, HOXB6, HOXB8, WNT3, FOXA2*) are down-regulated in these cells (Fig. 6c). *KDM5D* mutation-specific up-regulated genes participate in epithelial-mesenchymal transition, interferon response and importantly include 8 potential tumour suppressor genes (*IFI44L, DLG4, STRA6, GPRC5C, PTK7, NPTXR, BRINP1, SPINT1*), while several genes involved in cell-cell signalling including growth-factor receptors *FGFR3* and *PDGFRB* are down-regulated in these cells (Fig. 6d). The combination of coordinate upregulation of previously-described tumour suppressor genes and downregulation of proliferation-promoting growth factor receptors may plausibly account for the failure of these cells to form xenograft tumours. Further consistent with this idea, the up- and down-regulation of these genes were reversed by the co-mutation of *KDM5C* (Fig. 6d), which restores tumour formation by *KDM5D* mutant cells. Genes that were specifically upregulated in *KDM5C/KDM5D* mutant cells included long non-coding RNAs, while *EREG*, a gene belonging to the EGF family was downregulated (Fig. 6e). We reasoned that genes that were specifically dysregulated as a result of *KDM5C*, *KDM5D* or *KDM5C/KDM5D* mutation might also be dysregulated in *KDM5C* mutant human male ccRCC tumours, which also appear to have lost the Y chromosome and KDM5D function, however, this was not observed (Supplementary Fig. 4a–c). This result is also perhaps not surprising given the observation that *KDM5C* mutation had non-overlapping transcriptional consequences in the four tested ccRCC cell lines.

### KDM5C and KDM5D do not bind to promoters and enhancers of differentially expressed genes

**Fig. 7:**
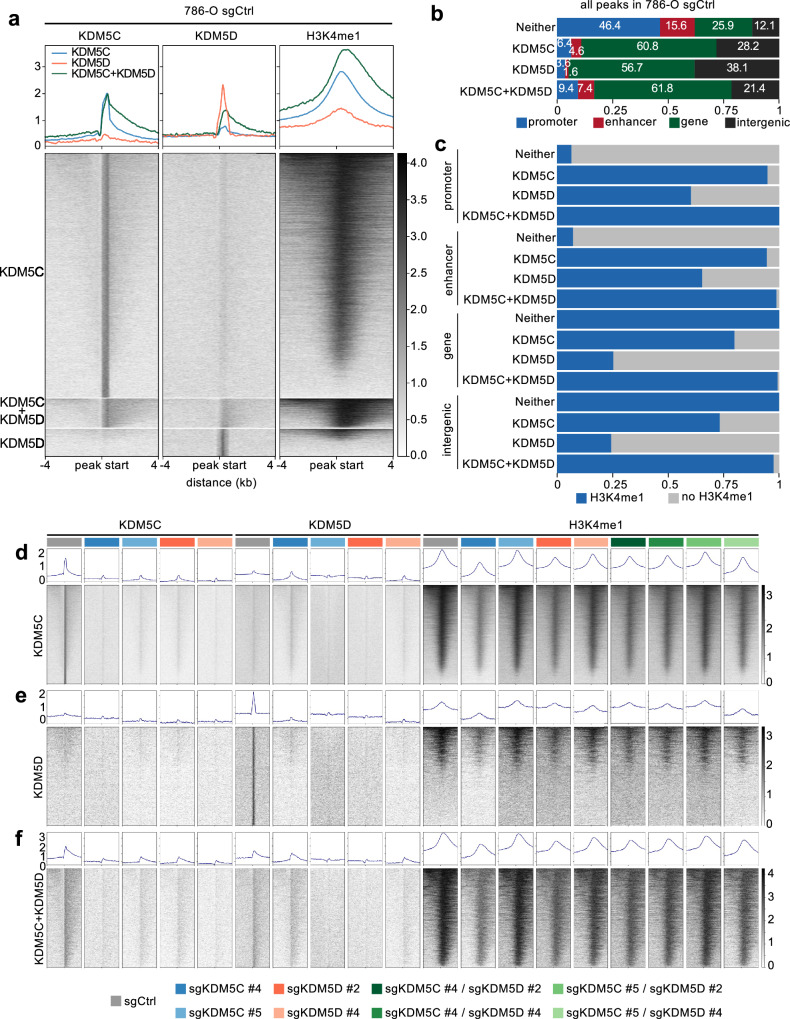
*KDM5C* and *KDM5D* mutations do not affect H3K4me1 in 786-O cells. **a** CUT&RUN KDM5C, KDM5D and H3K4me1 intensities at KDM5C and KDM5D binding sites in 786-O cells displayed as profiles (upper panels) and heatmaps (lower panels). **b** Percentage of genomic features with or without KDM5C, KDM5D or both KDM5C and KDM5D binding in 786-O cells. **c** Distribution of H3K4me1 at genomic features with or without detected KDM5C, KDM5D or both KDM5C and KDM5D binding (rows). **d**–**f** CUT&RUN signal for KDM5C, KDM5D and H3K4me1 as profiles (upper panels) and heatmaps (lower panels) in the indicated sgCtrl, sgKDM5C, sgKDM5D or sgKDM5C/sgKDM5D genotypes. Peaks are ordered by (**d**) all sites bound only by KDM5C, (**e**) all sites bound only by KDM5D, and (**f**) all sites bound by both KDM5C and KDM5D.

We next conducted CUT&RUN to identify KDM5C and KDM5D binding sites, as well as sites of enrichment of H3K4me1. The overlap of KDM5D binding sites with KDM5C binding sites in control cells was relatively high, considering that 52% of all KDM5D peaks directly overlapped with KDM5C peaks (Fig. 7a). However, it should be noted that many more peaks were identified as KDM5C than KDM5D binding sites, potentially consistent with the relatively higher mRNA expression of *KDM5C* than *KDM5D*. A potential caveat is that the relative activity and efficiency of each antibody in the CUT&RUN assay is unknown and it is possible that one antibody may under-represent the total number of binding sites of the protein due to a relative inefficiency in the assay. It is therefore possible that the true extent of overlapping binding is greater than observed. Almost all of the overlapping KDM5C and KDM5D peaks sites showed a co-occurring H3K4me1 signal which showed an overall increased intensity compared to sites with unique KDM5C or KDM5D binding. Interestingly, 73% of unique KDM5D binding sites had no co-occurring H3K4me1 signal, whereas only about 20% of unique KDM5C peaks did not overlap with H3K4me1 peaks. In general, these analyses demonstrate that both KDM5C and KDM5C occupy genomic regions that are also enriched in the histone mark that is generated by the enzymatic activity of these proteins.

In 786-O control cells, 6.4% of KDM5C peaks, 3.6% of KDM5D peaks, and 9.4% of KDM5C + KDM5D peaks were found at promoter coordinates. 4.6%, 1.6%, and 7.4% were bound at enhancer coordinates, respectively (Fig. 7b). The distribution of H3K4me1 at the different genomic features revealed that the relatively high number of KDM5C or KDM5D binding sites without H3K4me1 could mainly be associated to intergenic sites or sites within the gene body (Fig. 7c). Promoter and enhancer coordinates without KDM5C or KDM5D binding mainly showed no co-occurring H3K4me1 signal, while binding of KDM5C and/or KDM5D was mostly associated with H3K4me1 (Fig. 7c).

The intensity of KDM5C and KDM5D binding decreased in *KDM5C* or *KDM5D* mutant cells, respectively, as expected (Fig. 7d–f). Interestingly however, mutation of *KDM5D* reduced KDM5C binding to an extent similar to *KDM5C* mutation (Fig. 7d) and *KDM5C* mutation decreased KDM5D binding similarly to *KDM5D* mutation (Fig. 7e). This suggests that the binding of KDM5C or of KDM5D to their respective genomic sites is dependent on the presence of the other protein, indicative of an unexpected cooperativity of binding. However, even though the CUT&RUN anti-KDM5D antibody is specific in western blotting (Fig. 4d) (the CUT&RUN anti-KDM5C antibody does not work in western blotting) it cannot be excluded that there might be some antibody cross-reactivity in the CUT&RUN assays and that both antibodies can recognize both of the highly homologous KDM5C and KDM5D proteins, so the observed reductions in the peaks could also reflect a decrease in the total amount of KDM5C/KDM5D. In contrast to the effects of mutation of *KDM5C* in A498 cells which globally reduced H3K4me1 levels, there was no effect of either KDM5C or KDM5D single mutation, nor of *KDM5C/KDM5D* double mutation on H3K4me1 levels at any of the sites of binding of these proteins (Fig. 7d–f). It is possible that KDM5A or KDM5B may compensate for the loss of KDM5C/KDM5D function in 786-O cells, but less so in A498 cells. At least at the mRNA level, *KDM5A* and *KDM5B* are also expressed in all of the ccRCC cell lines used in this study (Supplementary Fig. 3).

Guided by the conclusions of the A498 CUT&RUN experiments, we next identified promoter and enhancer elements for all available *KDM5C* mutation-specific (Supplementary Fig. 5a), *KDM5D* mutation-specific (Supplementary Fig. 5b) or *KDM5C/KDM5D* mutation-specific (Supplementary Fig. 5c) up- or down-regulated genes. There was no enrichment for KDM5C or KDM5D binding at any of these sites, nor did mutation of *KDM5C* and/or *KDM5D* consistently induce changes in H3K4me1 at these sites (Supplementary Fig. 5a–c). We conclude that neither KDM5C nor KDM5D bind to the promoters or enhancers of genes whose expression is dysregulated by their mutation in 786-O cells, nor are they necessary for controlling H3K4me1 levels at these sites. The mechanism of gene regulation by KDM5C and KDM5D therefore appears to be different in 786-O cells to the previously proposed model of KDM5 enzyme family gene regulatory functions in other cells^22^.

## Discussion

The *KDM5C* gene is mutated in approximately 20% of ccRCC metastases but the molecular mechanisms through which loss of KDM5C function contribute to tumour evolution and metastatic spread remain incompletely characterised. While KDM5C functions as an H3K4 demethylase and is proposed to thereby regulate chromatin states and associated gene transcription, our findings argue that regulation of a specific and common gene expression pattern is very unlikely to be the mechanism through which KDM5C acts as a ccRCC tumour suppressor protein. *KDM5C* mutation in ccRCC cell lines altered the expression of only 100–200 genes and there were essentially no overlaps in the differentially expressed genes across four different ccRCC cell lines. This argument however does not imply that transcriptional alterations induced by *KDM5C* mutation do not contribute to ccRCC. It is possible that loss of KDM5C function alters gene expression in each individual tumour in combination with the constellation of genetic mutations and signalling pathway states that govern the overall transcriptional output in that tumour. This model is in keeping with KDM5C’s function as an enzyme that can demethylate H3K4 at many sites in the genome and thereby serve as an accessory enzyme to modulate gene expression driven by other factors, rather than itself being a direct driver of transcription. Consistent with this idea, a previous study using ES cells showed that in general, loss of KDM5C function alters the balance of H3K4me3 and H3K4me1 at promoters and enhancers to control the expression of some genes^22^. However, it should be noted that this effect was not black and white at the individual gene level. KDM5C bound to several thousand sites in the genome but the expression of only a few hundred genes were altered upon *KDM5C* knockdown. Genes near KDM5C binding sites could either be upregulated or downregulated upon *KDM5C* knockdown, even when segregated based on levels of H3K4me3 at those sites. In A498 and 786-O cells, we were unable to detect KDM5C binding to promoters and enhancers of the majority of genes whose expression is altered upon *KDM5C* mutation, suggesting that KDM5C might also impact gene expression through activities outside of promoters and enhancers, such as in gene bodies or in intergenic regions where KDM5C binding in A498 cells also correlates with enrichment of the H3K4me1 mark, which is produced as a result of KDM5 enzymatic activity. These regions may potentially also contain transcriptional regulatory elements that are controlled by the balance of H3K4 tri- and mono-methylation. It also cannot be excluded that secondary effects of *KDM5C* mutation dominate the overall contribution to the alteration of the transcriptome. The absence of histone demethylase activity of KDM5C in *KDM5C* mutant cells may also contribute to tumour evolution by inducing DNA replication stress^24^ or genomic instability^25^. Biochemical studies have also suggested that KDM5C and KDM5D might have numerous additional non-histone substrates that potentially extend the functions of KDM5 family enzymes beyond the regulation of histones and transcription^26^. While these putative substrates remain to be validated and functionally characterised, it is plausible that some of these proteins may also be relevant to ccRCC biology in *KDM5C* mutant and/or Y-chromosome loss tumours.

*KDM5C* was identified as an EXITS (Escape from X-chromosome inactivation tumour suppressor) gene^17^. This class of tumour suppressor genes are encoded on the X chromosome, are not silenced by X chromosome inactivation in female cells, are mutated more frequently in male than in female tumours and have a close homologue on the Y chromosome. In the case of *KDM5C*, the homologue is *KDM5D*. The strong enrichment of mutations in *KDM5C* in male ccRCC has been interpreted as being indicative of the absence of functional compensation by the Y chromosome-encoded homologue for the X chromosome-encoded tumour suppressor. Our observation that *KDM5C* mutation is accompanied by loss of the Y chromosome in most male ccRCC tumours is instead consistent with the idea that *KDM5C* and *KDM5D* may follow a modification of a classic Knudson two-hit tumour suppressor function that applies to sex chromosome gene homologues rather than alleles of the same gene. In keeping with the idea that KDM5C and KDM5D may have at least overlapping biological functions, KDM5D exhibits high amino acid similarity to KDM5C and both show equivalent histone demethylation activity in in vitro assays^26^. However, mouse genetic evidence showing that *Kdm5d* does not fully phenotypically compensate for the mutation of *Kdm5c* in male mice, as well as showing sex-specific and dosage-dependent effects of KDM5C on transcription, demonstrates that KDM5C and KDM5D have at least partly non-overlapping functions^27–31^. Our findings demonstrate that KDM5C and KDM5D have overlapping, as well as divergent functions in 786-O cells. While mutation of *KDM5C* or of *KDM5D* both induce the up- or down-regulation of a small set of common genes, the majority of differentially expressed genes are specific to either *KDM5C* or *KDM5D* mutation. Phenotypically, *KDM5C* and *KDM5D* mutations also had different effects. *KDM5C* mutation slightly reduced metastatic propensity, while *KDM5D* mutation completely blocked xenograft growth. Impressively, *KDM5C* co-mutation in *KDM5D* mutant cells completely restored tumour growth and metastasis. This was also observed at the transcriptional level where *KDM5D* mutation increased the expression levels of a series of putative tumour suppressor genes and decreased the expression of putative pro-proliferative genes, while *KDM5C* co-mutation reversed these transcriptional changes. We note that the reverse situation is not true; genes that are dysregulated following *KDM5C* mutation are not restored by *KDM5D* mutation. Thus, KDM5D gene regulatory functions are dependent on KDM5C, but not vice versa, at least in this cell type. The mechanism underlying this dependency is not clear as KDM5C or KDM5D do not bind to promoters or enhancers of the differentially expressed genes, nor did either single or double mutation of these genes in 786-O cells affect H3K4me1 levels at these sites, or globally. While we identified some overlapping binding sites between KDM5C and KDM5D, potentially consistent with either cooperative or antagonistic functions of the two proteins at certain genomic loci, this observation should be treated with caution due to the uncertainties surrounding the use of different antibodies with potentially different efficiencies and specificities in the CUT&RUN assay.

Nonetheless, the phenotypic data in 786-O cells are consistent with the observed genetics of human ccRCC which show that *KDM5C* mutation is almost always associated with a signature of low Y chromosome gene expression, suggestive of loss of the Y chromosome. By inference from 786-O cells, loss of the Y chromosome and *KDM5D* function may represent a barrier to further tumour evolution, which can be alleviated by *KDM5C* mutation, or vice versa. It will be important in future studies to investigate whether similar relationships between *KDM5C* and *KDM5D* mutations are observed in other male ccRCC cell lines that do not harbour *KDM5C* mutation and/or Y chromosome loss. It will also be interesting to determine whether complete loss of the Y chromosome is equivalent to *KDM5D* mutation, keeping in mind that the Y chromosome also harbours additional tumour suppressors such as *KDM6C*, the homologue of the EXITS tumour suppressor gene *KDM6A* which is mutated in rare cases of ccRCC^1^. We also note that the majority of ccRCC tumours with the Y chromosome loss gene expression signature do not harbour *KDM5C* mutations, arguing that there might be other mutations or cellular states that cooperate with the loss of the Y chromosome to permit or promote tumour evolution. However, our analyses of TCGA data revealed that there is no enrichment for other commonly recurrent ccRCC-specific gene mutations, including *PBRM1*, *BAP1*, *SETD2*, *ARID1A*, *TP53*, *PTEN* or *MTOR* in Y chromosome-deficient ccRCCs (data not shown).

Finally, this study highlights that there may be fundamental genetic differences between ccRCCs in men and in women and contributes to the growing understanding that Y chromosome loss is not just a bystander event during the formation of many different tumour types in men, but rather contributes in different ways to the evolution and pathology of these diseases, as well as to sex-specific differences in tumour incidence and biology^17,32,33^. For example, in bladder cancer, Y chromosome loss contributes to tumour immune evasion^23^ while in colorectal carcinoma, upregulation of *KDM5D* expression alters cell adhesion, antigen presentation and anti-tumour immunity, accounting for differences between male and female tumours^34^. These studies implicate Y chromosome status in crosstalk between tumour cells and immune cells. In this context it is notable that several genes involved in the interferon response are upregulated in *KDM5D* mutant 786-O cells, potentially reflecting activation of the STING-mediated immune response. STING activation induces the expression of several cytokines which can lead to the recruitment of different types of immune cells. Notably, *KDM5C* co-mutation abrogates the upregulation of the interferon-responsive genes in *KDM5D* mutant cells. Given that advanced ccRCC tumours are treated with immune checkpoint therapies, it will be important to further characterise the consequences of loss of the Y chromosome and mutation of *KDM5C* in the context of male ccRCC tumour evolution and tumour cell interactions with the immune system to determine if these different types of genetic alterations may shape the tumour immune microenvironment. In addition to analyses of human ccRCC tumours segregated based on Y chromosome and *KDM5C* mutation status, functional experiments introducing *Kdm5c* and *Kdm5d* mutations into mouse autochthonous ccRCC models could be informative.

## Methods

### Cell culture

Cell lines used in this study were 786-O (ATCC), 769-P (ATCC), A498 (ATCC), RCC4 (ECACC), SLR22 (from Holger Moch, University Hospital Zurich) and human primary RPTEC (ATCC). Cell lines were cultured in DMEM (Gibco) + 10% FCS (Sigma) + 1% Penicillin/Streptomycin (Gibco). Cell lines were routinely checked for mycoplasma contamination using PCR and their identities were validated by STS mapping and by exome sequencing and comparison of mutational profiles with public databases. MuLE lentiviruses expressing sgRNAs, spCas9 and either PuroR or NeoR were cloned and used to infect cells as previously described in ref. ^21^. sgRNA sequences (5’–3’) were sgCtrl (GTCATGTCACTTATCAAGTC), sgKDM5C #4 (Exon 8, CTGCCGGATGTGTTCTCGAG), sgKDM5C #5 (Exon 8, GTCTGCCGGATGTGTTCTCG), sgKDM5D #2 (Exon 2, TCAAAAACCGGGCACTCCGG), sgKDM5D#4 (Exon 4, CCATCTGCAAGGATCGTCGG).

### Western blotting

The following antibodies were used: anti-KDM5C (Bethyl Laboratories, A301-034A), anti-KDM5D (Bethyl Laboratories, A301-751), and anti-Vinculin (abcam, ab129002).

### Proliferation assay

Cells were plated at a density of 500 cells per well, in a 96-well plate, in 6 individual plates. After every second day, the medium of the plates was changed. All plates were subsequently (each 24 h after plating) fixed with 0.1% crystal violet (Sigma-Aldrich) plus 20% EtOH (SAV liquid production GmbH) in H_2_O for 12 min. Plates were rinsed three times with tap water and air-dried. All fixed and stained plates were resuspended with 0.5% Triton X-100 (Sigma-Aldrich) in 50% EtOH (SAV liquid production GmbH) and the absorbance was measured using Spark 10 M microplate reader (Tecan).

### Soft agar assay

A 2% low melting agarose (Sigma-Aldrich) bottom layer was diluted in a complete growth medium and plated, 5 × 10^4^ cells per well were diluted 1:1 in 0.4% low melting agarose in H_2_O. This mixture was added to the 2% bottom layer and allowed to solidify at room temperature for 30 min. Five hundred microliter medium was added on top to prevent matrix drying. The medium was changed every 72 h. After 4 weeks colonies were stained using 1 mg/mL Iodonitrotetrazoliumchloride (INT, Sigma-Aldrich) in DMSO (Sigma-Aldrich). INT solution was diluted 1:10 in complete growth medium and 500 µL were added to each well. Plates were incubated at 37 °C for around 18 h. Each individual well was documented via a stereo microscope (Zeiss) and visible colonies were counted manually.

### Xenograft tumour growth assays

Fresh medium was added to cells one day prior to injection. For harvesting, cells were washed once with PBS followed by detachment using StemPro Accutase (Gibco) for 5 min. StemPro Accutase was neutralized by adding a complete growth medium, followed by centrifugation (300 rpm, 4 min), 2 × 10^6^ cells per injection were resuspended in 75 µL serum-free Dulbecco’s Modified Eagle’s Medium (DMEM) or Roswell Park Memorial Institute (RPMI) (Gibco). All the following steps were performed on ice and with ice-chilled materials. A 30 G insulin syringe (BD) containing the following ingredients was prepared; first, 75 µL Matrigel Growth Factor Reduced (Corning) was added into the syringe, and on top of the Matrigel, 75 µL of the cell suspension was added. Matrigel and cell suspension were mixed within the syringe by flicking the syringe. For subcutaneous injection, 10-week-old SCID-beige mice were injected in a randomized, non-blinded manner. Female cell lines were injected into female mice and male cell lines were injected into male mice. Prior to injection, the mice were anaesthetized using isoflurane in O_2_ in an inhalation narcosis chamber. The right flank was shaved, and the skin was disinfected with 70% ethanol (EtOH). The components of the syringe were again mixed, and the needle tip was also disinfected shortly before the injection. The cells were injected subcutaneously in the right flank of the mice. After the injection of the tumour cells, the injection site was cleaned and again disinfected. The tumour volume was monitored every week by measuring length and width with a caliper and tumour volume was calculated based on the following formula: volume = (length × width^2^)/2. Every second week, tumour formation is analysed using non-invasive in vivo bioluminescence imaging (BLI). Mice were anaesthetized using vaporized isoflurane in O_2_ in an inhalation narcosis chamber and subcutaneously injected with 150 mg/kg XenoLight D-luciferin (Perkin Elmer). After 12 min, bioluminescence was recorded using IVIS Lumina III (PerkinElmer) and analysed with the associated Living Image software (version 4.5). Shortly before sacrifice, measurement of tumour volume and BLI were repeated and 10 min after injection, mice were sacrificed by cervical dislocation and primary tumours, as well as relevant organs (lung, liver, spleen, kidneys, femur and brain) were dissected. From this point on, primary tumours and organs were kept in sterile PBS on ice. All dissected tumours and organs were imaged with BLI. No animals were excluded from the analyses. We have complied with all relevant ethical regulations for animal use. Animal experiments were conducted under licence G17-165 approved by the Regierungspräsidium Freiburg, Germany. The maximum volume of tumours allowed under this experimental licence was 1 cm^3^ and in none of the experiments was this volume exceeded.

### RNA-seq

RNA was isolated from cells using the NucleoSpin RNA Mini kit (Macherey-Nagel). Paired-end RNA-sequencing was performed on an Illumina NovaSeq 6000 System (flow cell type S1 for 2 × 100 bp) by the core facility of the DKFZ (German Cancer Research Center) in Heidelberg with the Illumina TruSeq Stranded RNA library preparation kit. Read trimming, alignment, normalization, log2 transformation and differential gene expression were conducted as previously described^19^. Genes with a sum of raw counts in all corresponding samples less than 60 (KDM5C analyses) or 120 (KDM5C/KDM5D analyses) were removed. Supporting Data Files 1 and 2 contain log_2_FC, normCounts and rlogCounts for the different datasets. Raw RNA sequencing data has been uploaded to GEO with the identifiers GSE284611 (*KDM5C* mutation) and GSE284610 (*KDM5C/KDM5D* mutations).

### CUT&RUN sample processing

One million cells were harvested, washed, and bound to 10 µL activated Concavilin A-coated magnetic beads (Bangs Laboratories, 86057) and then permeabilized in 0,03% Digitonin-containing buffer (20 mM HEPES, 150 mM NaCl, 0.5 mM Spermidin, 1 Roche Protease Inhibitor Cocktail Tablet/50 mL). The cell-bead slurry was incubated with 50 µL antibody buffer (2 mM EDTA, 1:100 antibody dilution in Digitonin Buffer) on a nutator overnight at 4 °C. One of the following antibodies were added to the appropriate reaction: IgG negative control (Cell Signalling Technology, #58802), anti-KDM5C (Abcam, ab34718), anti-KDM5D (BethylLab A301-751A), anti-H3K4me1 (Epicypher, 13-0040), anti-H3K4me3 (ActiveMotif, 39159), anti-H3K27ac (Epicypher, 13-0045), anti-H3K27me3 (Cell Signalling Technology, #9733). The beads were then washed and resuspended in 100 µL Digitonin buffer containing 700 ng/mL ProtA/G-MNAse (micrococcal nuclease) fusion protein and incubated on a nutator for 1 h at 4 °C. The beads were later washed twice and incubated on ice for 5 min. Three mcroliter of ice-cold 100 mM CaCl_2_ was added to the beads, the mixture was gently vortexed and incubated on ice for 30 min. After the incubation time, 100 µL STOP buffer (340 mM NaCl, 20 mM EDTA, 4 mM EGTA, 0.05% Digitonin, 50 µg/mL RNAse A, 50 µg/mL Glycogen) containing 1 ng *Escherichia coli* or *Drosophila melanogaster* fragmented DNA as spike-in control (EpiCypher, 18-1401) was added. The mixture was gently vortexed and incubated at 37 °C for 30 min. Beads were then centrifuged at 16.000 *g* for 5 min, the protein-DNA fragments were released in the supernatant and DNA was extracted followed by library preparation with NEBNext® Ultra™ II DNA Library Prep Kit and NEBNext® Multiplex Oligos Set (New England Biolabs) without size selection. The DNA concentration and size distribution of library fragments were determined via Qubit and Agilent 2200 TapeStation, respectively. The libraries were subjected to paired-end sequencing on Illumina NextSeq 500/550 (mid output kit v2.5 for 2 × 75 bp), MiSeq (reagent kit v3 for 2 × 75 bp), or NovaSeq 6000 (flow cell type SP for 2 × 100 bp) systems in the core facility of the DKFZ in Heidelberg. Raw and processed CUT&RUN sequencing data has been uploaded to GEO with the identifier GSE284609.

### CUT&RUN bioinformatic data processing

Raw FASTQ files were subjected to quality control and adaptors and low-quality ends were trimmed using Trim Galore (version 0.6.6) with default settings. The alignment was performed using Bowtie2 (version 2.4.5)^35^. The human reads were aligned to the UCSC hg19 reference genome and the spike-in control accordingly to the *D. melanogaster* UCSC dm6 reference genome for A498 experiments or *E. coli* NCBI K12 MG1655 reference genome for 786-O experiments. Based on the resulting BAM files, BigWig files were generated using bamCoverage from deepTools (version 3.5.0)^36^ which can be used to visualize the coverage via the Integrative Genomics Viewer (IGV)^37^. Additionally, for each sample, including the IgG control, the total number of aligned reads for both human and spike-in were calculated by samtools (version 1.16.1) flagstat^38^ and the reads were normalized. First, the ratio of spike-in (*spike*) and human (*genome*) reads was calculated for each experimental sample (*expr*) (formula 1) and the IgG control (ctrl) (formula 2). Subsequently, a size factor was calculated by dividing the ratio of the control by the ratio of the experimental sample (formula 3). While the normalized reads for the experimental sample simply are the total human reads (*exprgenome*), the control is normalized for each experimental sample by multiplying the appropriate size factor with the total human reads from the IgG control (*ctrlgenome*) (formula 4) resulting in *norm_ctrlgenome*.

1 $${{ratio}}_{{expr}}=\frac{{{expr}}_{{spike}}}{{{expr}}_{{genome}}}$$

2 $${{ratio}}_{{ctrl}}=\frac{{{ctrl}}_{{spike}}}{{{ctrl}}_{{genome}}}$$

3 $${size}{{\_}}{factor}=\frac{{{ratio}}_{{ctrl}}}{{{ratio}}_{{expr}}}$$

4 $${{norm}{{\_}}{ctrl}}_{{genome}}={size}{{\_}}{factor}* {{ctrl}}_{{genome}}$$

These normalized reads were used to generate tag directories using makeTagDirectory with the option -totalReads from Homer (Hypergeometric Optimization of Motif Enrichment) (version 4.11)^39^ which parses through the alignment file and splits the tags into separate files based on the chromosomes. These tag directories were further used for computing coverage and peak calling.

For A498 cells treated with H3K27ac antibodies, only a few peaks were detected based on the previously described normalization method. To this aim, another normalization method was used for the affected samples which resulted in a higher number of peaks for downstream analyses. For both the experimental and control samples, the mapped reads (*samplegenome*) were first normalized to the number of initially anticipated reads per sample (formula 5), as well as to the sum of reads for all samples (formula 6), resulting in formula 7. The ratio of mapped reads to the genome (formula 8), as well as to the spike-in (formula 9) was calculated by dividing each sample by the control. Finally, formula 10 resulted in the normalized reads for each sample used to generate tag directories.

5 $${sampl}{e}_{{totalReads}}=\frac{{sampl}{e}_{{aimedReads}}}{{sampl}{e}_{{totalReadsProcessed}}}$$

6 $${al}{l}_{{totalReads}}=\frac{{al}{l}_{{aimedReads}}}{{al}{l}_{{totalReadsProcessed}}}$$

7 $${nor}{m}_{{totalReads}}={sampl}{e}_{{genome}}* \frac{{sampl}{e}_{{totalReads}}}{{al}{l}_{{totalReads}}}$$

8 $${rati}{o}_{{genome}}=\frac{{sampl}{e}_{{genome}}}{{ctr}{l}_{{genome}}}$$

9 $${rati}{o}_{{spike}}=\frac{{sampl}{e}_{{spike}}}{{ctr}{l}_{{spike}}}$$

10 $${norm}{{\_}}{sampl}{e}_{{genome}}=\frac{{nor}{m}_{{totalReads}}}{\frac{{rati}{o}_{{genome}}}{{rati}{o}_{{spike}}}}$$

### CUT&RUN peak calling

Peaks were called using Homer’s findPeaks function using the histone mode with default parameters. The option -C was set to 0 to disable filtering based on clonal signals, since a high frequency of fragments with identical ends can generally arise from different cells in CUT&RUNseq experiments. Experimental samples were directly compared to the IgG background control and only peaks with 4-fold more tags in the experimental sample compared to the control (sequencing-depth independent), as well as with a cumulative Poisson *p*-value ≤ 0.0001 (sequencing depth dependent) were kept. Peaks within blacklisted regions from ENCODE^40^, such as repetitive sequences, were excluded using intersectBed from BEDTools (version 2.27.1)^41^. The peak calling analysis results in a bed file with all identified peaks with their genomic location and several statistics.

In order to explore the peaks, unique and overlapping peaks were identified between the different samples using Homer mergePeaks with the distance option -d given. This option requires the peaks to have a specific overlap between the start and end coordinates in order to be merged. For visualizations of the signal intensity around peaks or other genomic locations, heatmaps were generated using functions from deepTools. First, bamCompare was used to compare the read counts of each experimental sample to the IgG control with the option—scale factors 1:S, whereas S is equal to *ratio*_*expr*_ (formula 1 above) divided by *ratio*_*ctrl*_ (formula 2 above). The resulting signal intensities are represented as the log2 of the ratio of the sequencing depth normalized reads. Next, computeMatrix was used to generate a matrix of signal intensities up- and downstream of the peak centres which was subjected to plotHeatmap and plotProfile for visualizations.

### CUT&RUN promoter and enhancer analyses

In order to identify promoter and enhancer coordinates, public annotation databases were used. The RefSeq hg19 database was used to identify promoter-gene pairs. Enhancer-gene pairs were retrieved from the GeneHancer hg19^42^ database (version v5.17) for kidney tissues. Gene coordinates were retrieved from biomart GRCh37.p13 based on Ensembl IDs. In order to determine transcriptional states of genomic features, coordinates from genes, promoters and enhancers were overlapped with histone marks. Enhancers overlapping with promoters were removed. For each gene, the unique transcriptional state of its promoter and available enhancer was selected.

### Analyses of TCGA KIRC data

Publicly available raw RNA-seq data of the TCGA (The Cancer Genome Atlas) KIRC (Kidney renal clear cell carcinoma) samples were downloaded and processed as previously described^19^ and used to generate gene expression heatmaps. cBioportal^43,44^ was used to investigate the expression of Y chromosome genes in relation to *KDM5C* mutation status.

### Statistics and reproducibility

In addition to the statistical tests described in the bioinformatic analyses above, this study employed the two-sided Student’s unpaired *t*-test with Welch’s correction and one-way ANOVA with Dunnett’s multiple comparisons test. *p* < 0.05 was considered statistically significant.

### Reporting summary

Further information on research design is available in the Nature Portfolio Reporting Summary linked to this article.

## Supplementary information

Supplementary Information

Description of Additional Supplementary File

Supplementary Dataset 1

Supplementary Dataset 2

Supplementary Dataset 3

Supplementary Dataset 4

Reporting Summary

Peer Review

## Data Availability

Raw RNA sequencing data has been uploaded to GEO with the identifiers GSE284611 (*KDM5C* mutation) and GSE284610 (*KDM5C/KDM5D* mutations). Raw and processed CUT&RUN sequencing data has been uploaded to GEO with the identifier GSE284609. Supplementary Data File 1 contains normalized and normalized log2 transformed counts of RNA-seq data from *KDM5C* mutant cells. Supplementary Data File 2 contains normalized and normalized log2 transformed counts of RNA-seq data from the *KDM5C/KDM5D* single and double mutation experiments. Supplementary Data File 3 contains lists of differentially expressed genes from the *KDM5C/KDM5D* single and double mutation experiments. Supplementary Data File 4 contains source data for all graphs. Uncropped western blots are provided in Supplementary Fig. 6. All other data are available from the corresponding author on reasonable request.
