# Supplementary material for: KDM5C and KDM5D mutations have different consequences in clear cell renal cell carcinoma cells
Source: Commun Biol. 2025 Feb 15;8:244. doi: 10.1038/s42003-025-07695-8 (PMC11830100; doi:10.1038/s42003-025-07695-8)
Supplement: Supplementary file 1 — Supplementary Information [file 42003_2025_7695_MOESM1_ESM.pdf]

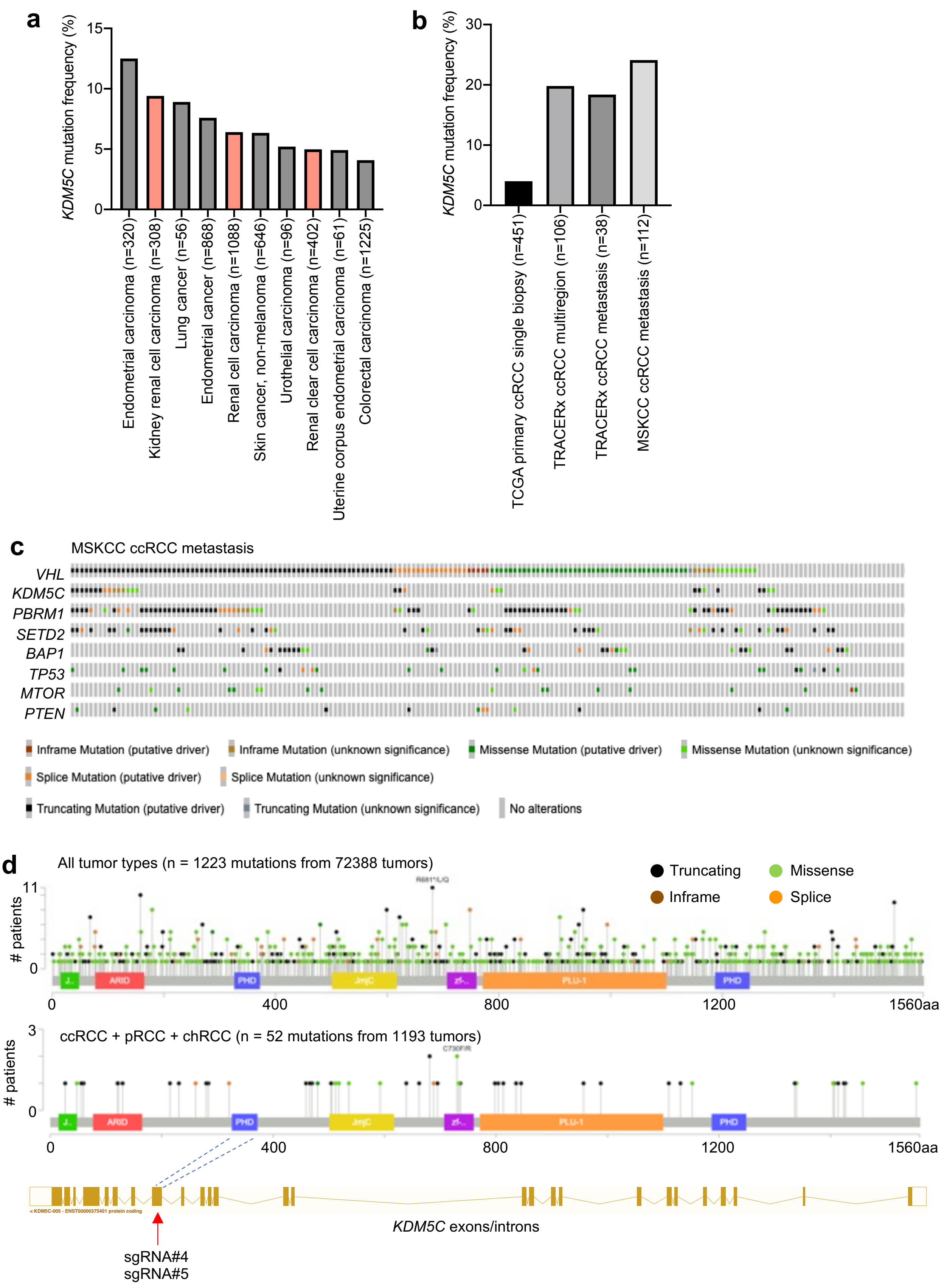

Supplementary Fig. 1: Mutations of *KDM5C* in human ccRCC.
a,b Frequency of *KDM5C* mutations in the indicated studies. Data derived from cBioPortal or from the studies cited in the Results section. **c** Oncoprint of the indicated mutations in ccRCC studies from the MSKCC metastasis sequencing cohort. Data derived from cBioPortal. **d** Types of *KDM5C* mutations across all human tumors or in RCC tumours (data derived from cBioPortal) mapped onto the protein domain structure of KDM5C with the corresponding exon/intron structure of the *KDM5C* gene. The two sgRNAs targeting exon 8 used in this study are depicted.

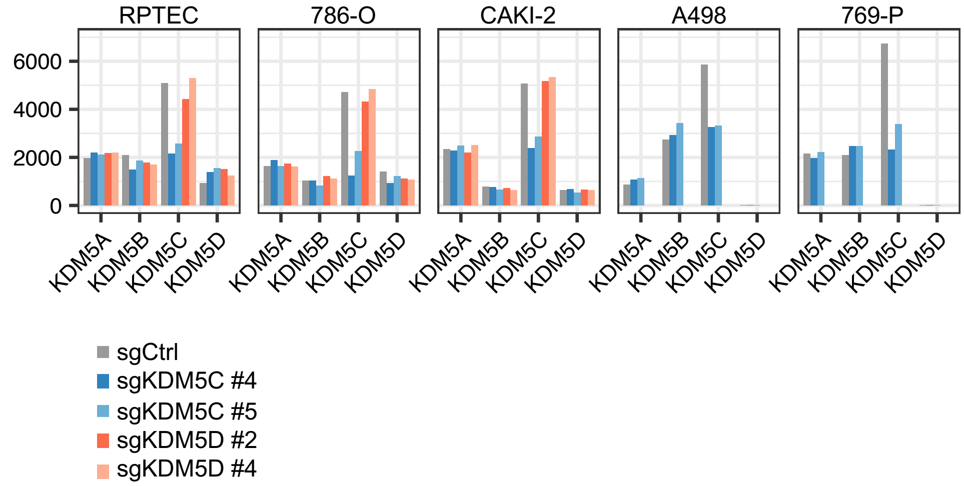

Supplementary Fig. 2: Expression levels of KDM5 family genes.
Normalised counts deriving from DESeq2 indicate the gene expression levels of *KDM5A*, *KDM5B*, *KDM5C* and *KDM5D* in the indicated cell lines infected with the indicated sgKDM5C or sgKDM5D.

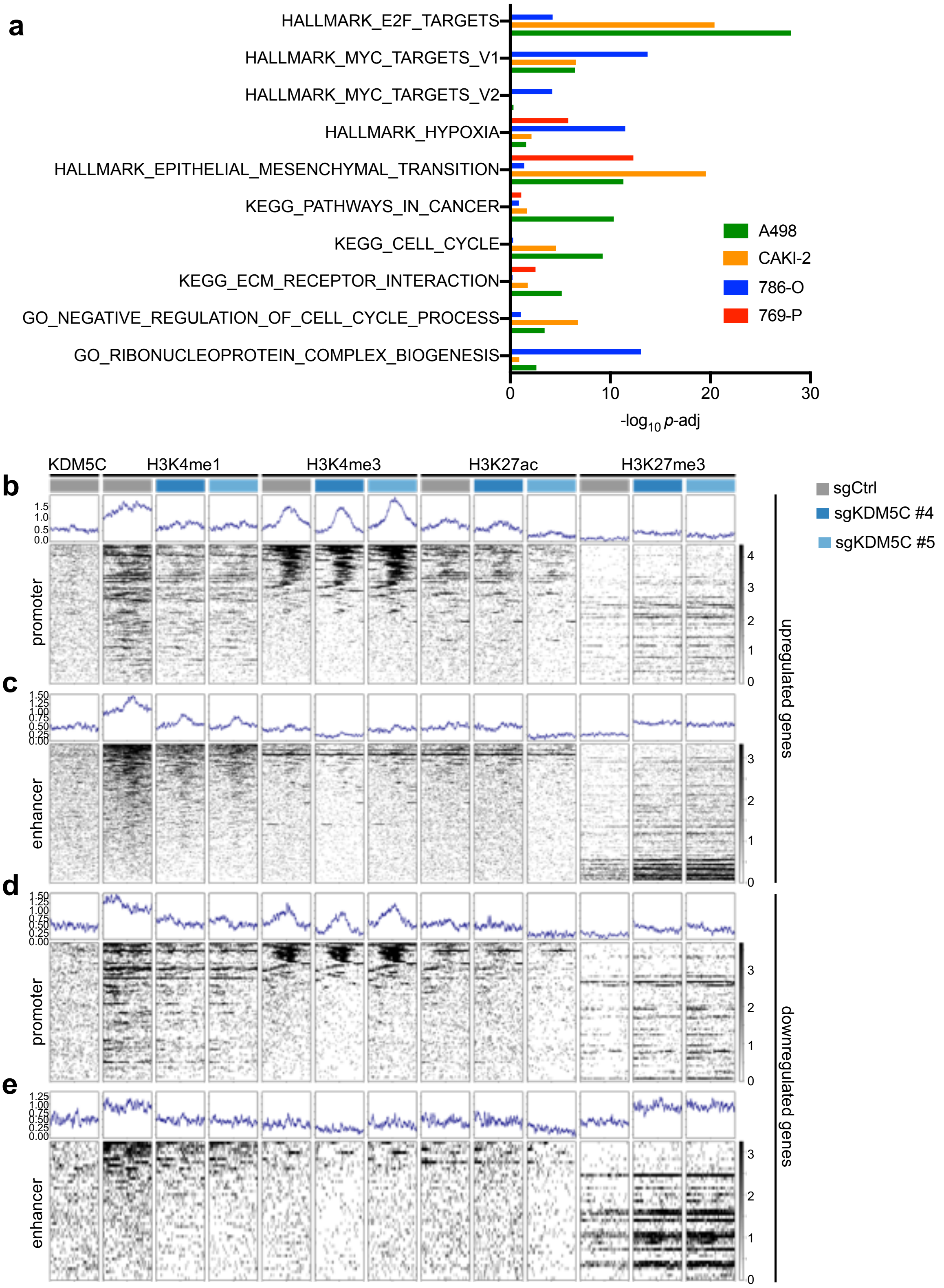

Supplementary Fig. 3: Absence of binding of KDM5C to promoter or enhancer elements of KDM5C-regulated genes.
a Selected gene set enrichment terms that are upregulated in *KDM5C* mutant cells. **b-e** CUT&RUN KDM5C, H3K4me1, H3K4me3, H3K27ac and H3K27me3 intensities in sgCtrl, sgKDM5C #4 and sgKDM5C #5 A498 cells displayed as profiles (upper panels) and heatmaps (lower panels). Genomic loci with 4kb up-and downstream regions representing (**b**) promoter elements and (**c**) enhancer elements of genes upregulated in *KDM5C* mutant cells and (**d**) promoter elements and (**e**) enhancer elements of genes downregulated in *KDM5C* mutant cells.

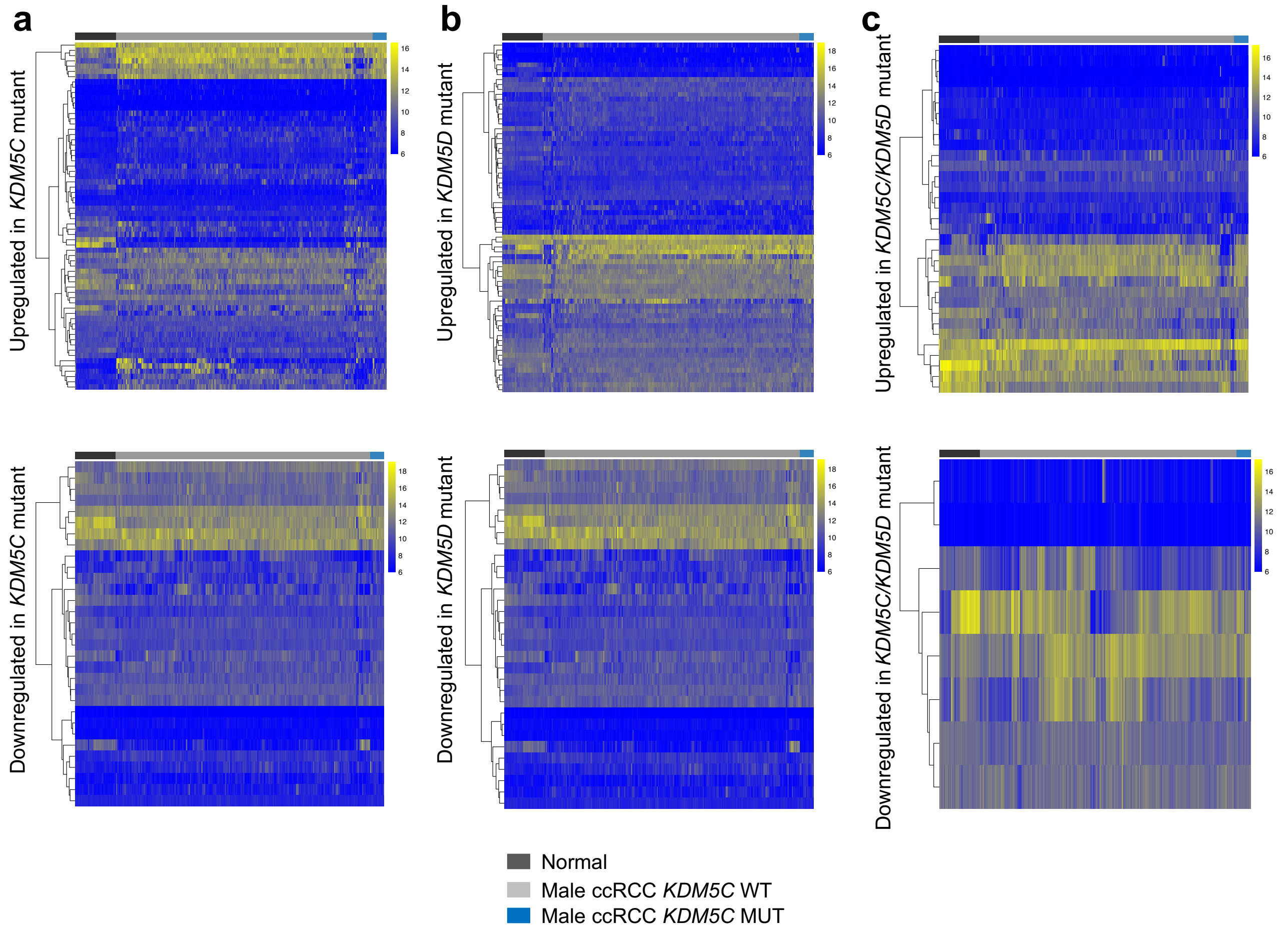

Supplementary Fig. 4: a-c Heatmaps of TGCA samples of normal kidney, *KDM5C* wild type ccRCC and *KDM5C* mutant ccRCC showing expression levels (z-scores) of genes that were identified in Figure 6 C-E as being dysregulated in (**a**) *KDM5C* mutant, (**b**) *KDM5D* mutant or (**c**) *KDM5C/KDM5D* double mutant 786-O cells.

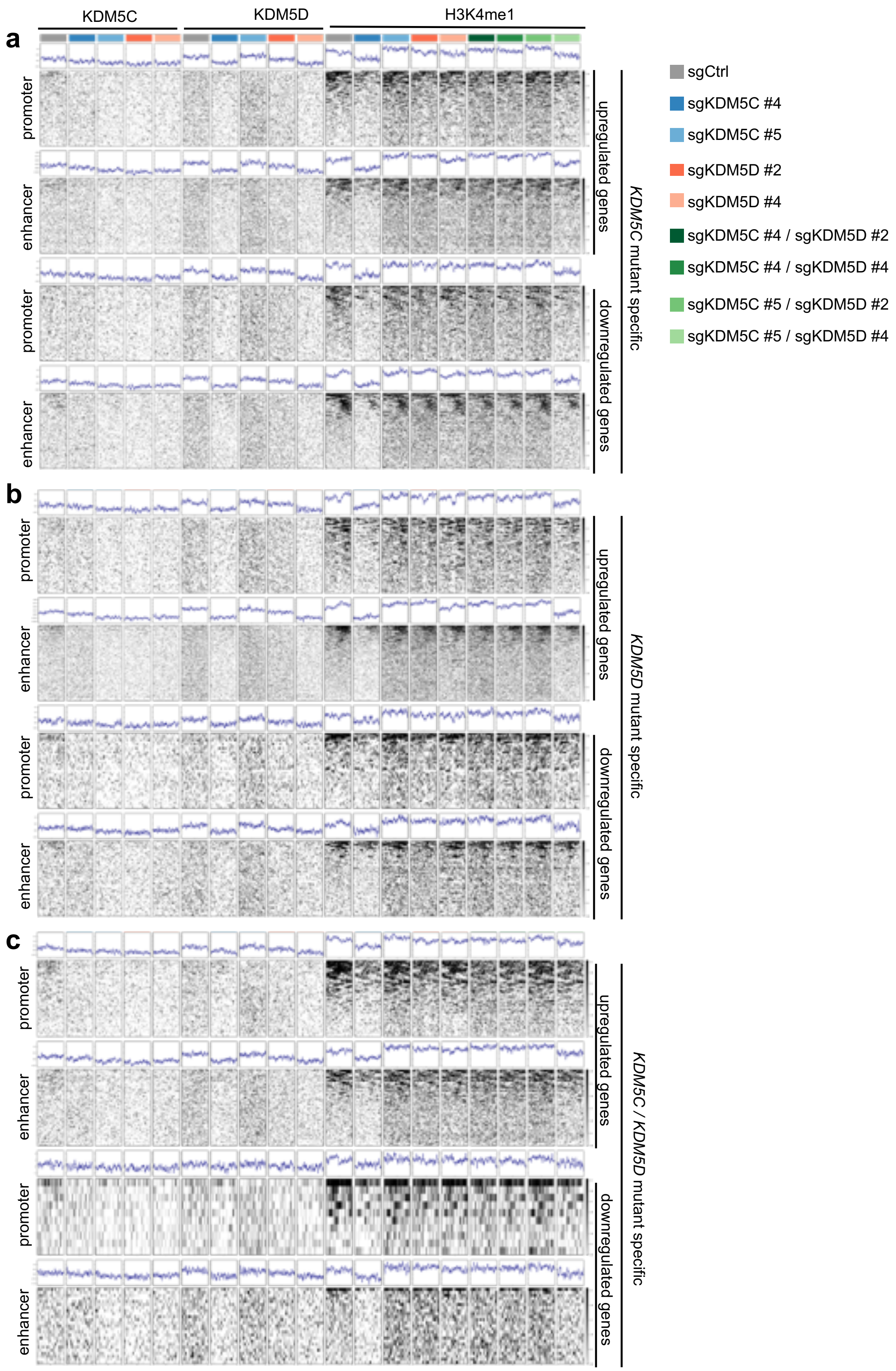

Supplementary Fig. 5: Absence of binding of KDM5C or KDM5D to promoter or enhancer elements of KDM5C- or KDM5D-regulated genes.
a-c CUT&RUN KDM5C, KDM5D and H3K4me1 intensities in sgCtrl or the indicated sgKDM5C and sgKDM5D genotypes of 786-O cells displayed as profiles (upper panels) and heatmaps (lower panels). Genomic loci with 4kb up- or downstream represent (**a**) promoter elements and enhancer elements of genes that are up- or downregulated in *KDM5C* mutant cells, (**b**) promoter elements and enhancer elements of genes that are up- or downregulated in *KDM5D* mutant cells and (**c**) promoter elements and enhancer elements of genes that are up- or downregulated in *KDM5C/KDM5D* mutant cells.

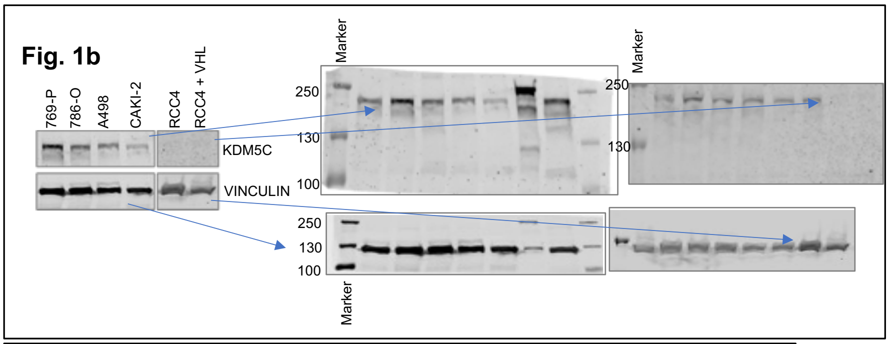

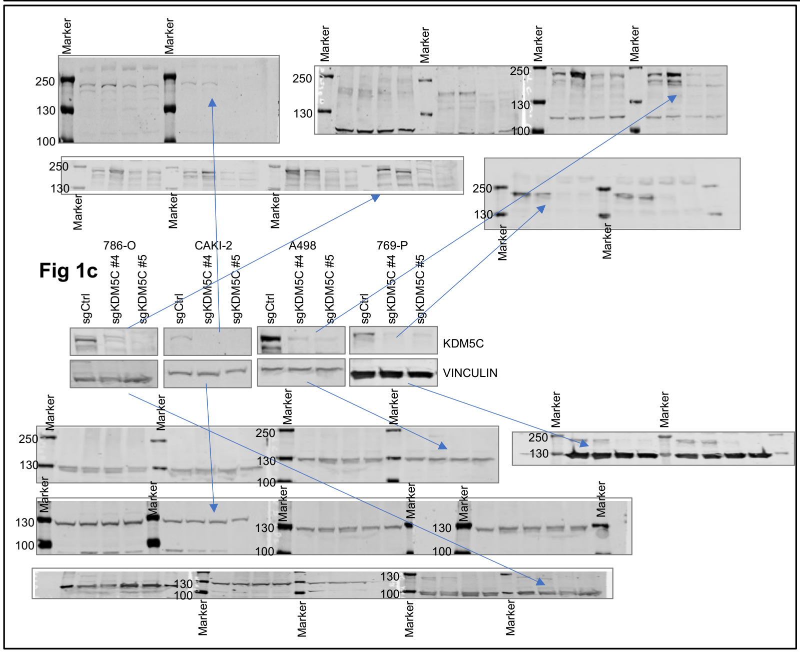

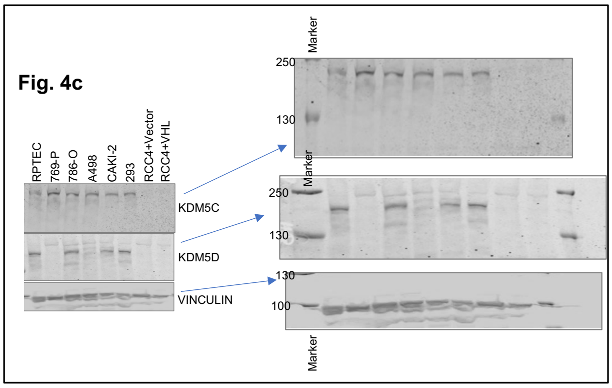

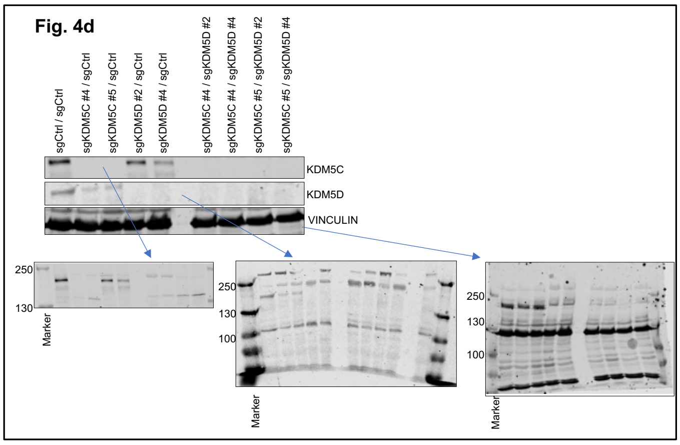

Supplementary Fig. 6: Full western blot scans

Full scans of western blots depicted in Fig. 1b, Fig. 1c, Fig. 4c and Fig. 4d. Lanes with marker proteins (masses in kDa are provided) are indicated on each blot.
