# Supplementary material for: KDM5C and KDM5D mutations have different consequences in clear cell renal cell carcinoma cells
Source: Commun Biol. 2025 Feb 15;8:244. doi: 10.1038/s42003-025-07695-8 (PMC11830100; doi:10.1038/s42003-025-07695-8)
Supplement: Supplementary file 2 — Description of Additional Supplementary File [file 42003_2025_7695_MOESM2_ESM.pdf]

# Description Of Additional Supplementary Data

**File name:** Supplementary Data File 1
**Description:** normalized and normalized log2 transformed counts of RNA-seq data from *KDM5C* mutant cells.

**File name:** Supplementary Data File 2
**Description:** normalized and normalized log2 transformed counts of RNA-seq data from the *KDM5C/KDM5D* single and double mutation experiments.

**File name:** Supplementary Data File 3
**Description:** differentially expressed genes from the *KDM5C/KDM5D* single and double mutation experiments.

**File name:** Supplementary Data File 4
**Description:** source data for all graphs.
