# Supplementary material for: KDM5C and KDM5D mutations have different consequences in clear cell renal cell carcinoma cells
Source: Commun Biol. 2025 Feb 15;8:244. doi: 10.1038/s42003-025-07695-8 (PMC11830100; doi:10.1038/s42003-025-07695-8)
Supplement: Supplementary file 7 — Reporting Summary [file 42003_2025_7695_MOESM7_ESM.pdf]

nature portfolio

Corresponding author(s): Ian Frew

Last updated by author(s): Jan 8, 2025

Reporting Summary

Nature Portfolio wishes to improve the reproducibility of the work that we publish. This form provides structure for consistency and transparency in reporting. For further information on Nature Portfolio policies, see our Editorial Policies and the Editorial Policy Checklist.

Statistics

For all statistical analyses, confirm that the following items are present in the figure legend, table legend, main text, or Methods section.

n/a	Confirmed	
☐	☒	The exact sample size (*n*) for each experimental group/condition, given as a discrete number and unit of measurement
☒	☐	A statement on whether measurements were taken from distinct samples or whether the same sample was measured repeatedly
☐	☒	The statistical test(s) used AND whether they are one- or two-sided<br>*Only common tests should be described solely by name; describe more complex techniques in the Methods section.*
☒	☐	A description of all covariates tested
☐	☒	A description of any assumptions or corrections, such as tests of normality and adjustment for multiple comparisons
☐	☒	A full description of the statistical parameters including central tendency (e.g. means) or other basic estimates (e.g. regression coefficient) AND variation (e.g. standard deviation) or associated estimates of uncertainty (e.g. confidence intervals)
☐	☒	For null hypothesis testing, the test statistic (e.g. *F*, *t*, *r*) with confidence intervals, effect sizes, degrees of freedom and *P* value noted<br>*Give P values as exact values whenever suitable.*
☒	☐	For Bayesian analysis, information on the choice of priors and Markov chain Monte Carlo settings
☒	☐	For hierarchical and complex designs, identification of the appropriate level for tests and full reporting of outcomes
☒	☐	Estimates of effect sizes (e.g. Cohen's *d*, Pearson's *r*), indicating how they were calculated

Our web collection on statistics for biologists contains articles on many of the points above.

Software and code

Policy information about availability of computer code

Data collection: No software was used

Data analysis: No software was used

For manuscripts utilizing custom algorithms or software that are central to the research but not yet described in published literature, software must be made available to editors and reviewers. We strongly encourage code deposition in a community repository (e.g. GitHub). See the Nature Portfolio guidelines for submitting code & software for further information.

Data

Policy information about availability of data

All manuscripts must include a data availability statement. This statement should provide the following information, where applicable:

- Accession codes, unique identifiers, or web links for publicly available datasets
- A description of any restrictions on data availability
- For clinical datasets or third party data, please ensure that the statement adheres to our policy

This information has all been included in the Materials section of the manuscript but is provided along with the relevant methods, rather than in a separate data availabiluty statement. GEO identifiers of RNA-seq and CUT&RUN datasets are included.

Research involving human participants, their data, or biological material

Policy information about studies with human participants or human data. See also policy information about sex, gender (identity/presentation), and sexual orientation and race, ethnicity and racism.

Reporting on sex and gender	Human cell lines were analysed based on sex
Reporting on race, ethnicity, or other socially relevant groupings	*Please specify the socially constructed or socially relevant categorization variable(s) used in your manuscript and explain why they were used. Please note that such variables should not be used as proxies for other socially constructed/relevant variables (for example, race or ethnicity should not be used as a proxy for socioeconomic status).* *Provide clear definitions of the relevant terms used, how they were provided (by the participants/respondents, the researchers, or third parties), and the method(s) used to classify people into the different categories (e.g. self-report, census or administrative data, social media data, etc.)* *Please provide details about how you controlled for confounding variables in your analyses.*
Population characteristics	*Describe the covariate-relevant population characteristics of the human research participants (e.g. age, genotypic information, past and current diagnosis and treatment categories). If you filled out the behavioural & social sciences study design questions and have nothing to add here, write "See above."*
Recruitment	*Describe how participants were recruited. Outline any potential self-selection bias or other biases that may be present and how these are likely to impact results.*
Ethics oversight	*Identify the organization(s) that approved the study protocol.*

Note that full information on the approval of the study protocol must also be provided in the manuscript.

Field-specific reporting

Please select the one below that is the best fit for your research. If you are not sure, read the appropriate sections before making your selection.

☒ Life sciences ☐ Behavioural & social sciences ☐ Ecological, evolutionary & environmental sciences

For a reference copy of the document with all sections, see nature.com/documents/nr-reporting-summary-flat.pdf

Life sciences study design

All studies must disclose on these points even when the disclosure is negative.

Sample size	Sample sizes were not predetermined as effect sizes were not known
Data exclusions	No data were excluded
Replication	Replication of cell culture experiments were successful. Pooled data from multiple experiments are depicted.
Randomization	Samples were not randomised as all experiments involved direct comparisons of wild type and mutant cells
Blinding	Blinding was not relevant to the study as all experiments involved direct comparisons of wild type and mutant cells in assays with defined quantitative outputs that could not be influenced by the investigator.

Reporting for specific materials, systems and methods

We require information from authors about some types of materials, experimental systems and methods used in many studies. Here, indicate whether each material, system or method listed is relevant to your study. If you are not sure if a list item applies to your research, read the appropriate section before selecting a response.

Materials & experimental systems

n/a	Involved in the study	
☐	☒	Antibodies
☐	☒	Eukaryotic cell lines
☒	☐	Palaeontology and archaeology
☐	☒	Animals and other organisms
☒	☐	Clinical data
☒	☐	Dual use research of concern
☒	☐	Plants

Methods

n/a	Involved in the study	
☐	☒	ChIP-seq
☒	☐	Flow cytometry
☒	☐	MRI-based neuroimaging

Antibodies

Antibodies used	Western Blotting: The following antibodies were used: anti-KDM5C (Bethyl Laboratories, A301-034A), anti-KDM5D (Bethyl Laboratories, A301-751), anti-VINCULIN (abcam, ab129002).<br>CUT&RUN: One of the following antibodies were added to the appropriate reaction: IgG negative control (Cell Signaling Technology, #58802), anti-KDM5C (Abcam, ab34718), anti-KDM5D (BethylLab A301-751A), anti-H3K4me1 (Epicypher, 13-0040), anti-H3K4me3 (ActiveMotif, 39159), anti-H3K27ac (Epicypher, 13-0045), anti-H3K27me3 (Cell Signaling Technology, #9733).
Validation	Descriptions of the validation of all antibodies used in this study are available on the manufacturer's websites and associated antibody datasheets. In many cases, our genetic knockout and knockdown studies described in this manuscript further validate the specificity of these antibodies.

Eukaryotic cell lines

Policy information about cell lines and Sex and Gender in Research

Cell line source(s)	Cell lines used in this study were 786-O, 769-P, A498 (all ATCC), RCC4 (ECACC), SLR22 (from Holger Moch, University Hospital Zurich) and human primary RPTEC (ATCC).
Authentication	Cell lines were validated by mutational calling from Exome-seq and RNA-seq data and matched against mutations described for these cell lines in publicly available databases.
Mycoplasma contamination	All cells tested negative for mycoplasma (PCR testing)
Commonly misidentified lines<br>(See ICLAC register)	None

Animals and other research organisms

Policy information about studies involving animals; ARRIVE guidelines recommended for reporting animal research, and Sex and Gender in Research

Laboratory animals	10-week-old SCID-beige mice
Wild animals	The study did not involve wild animals
Reporting on sex	Sex was considered in the study design and male and female ccRCC cell lines were analysed separately
Field-collected samples	The study did not involve samples collected from the field.
Ethics oversight	Mouse tumour xenograft experiments were conducted under experimental license G-17/165 of the Regierungspräsidium Freiburg

Note that full information on the approval of the study protocol must also be provided in the manuscript.

Plants

Seed stocks	*Report on the source of all seed stocks or other plant material used. If applicable, state the seed stock centre and catalogue number. If plant specimens were collected from the field, describe the collection location, date and sampling procedures.*
Novel plant genotypes	*Describe the methods by which all novel plant genotypes were produced. This includes those generated by transgenic approaches, gene editing, chemical/radiation-based mutagenesis and hybridization. For transgenic lines, describe the transformation method, the number of independent lines analyzed and the generation upon which experiments were performed. For gene-edited lines, describe the editor used, the endogenous sequence targeted for editing, the targeting guide RNA sequence (if applicable) and how the editor was applied.*
Authentication	*Describe any authentication procedures for each seed stock used or novel genotype generated. Describe any experiments used to assess the effect of a mutation and, where applicable, how potential secondary effects (e.g. second site T-DNA insertions, mosiacism, off-target gene editing) were examined.*

Data deposition

☒ Confirm that both raw and final processed data have been deposited in a public database such as GEO.

☒ Confirm that you have deposited or provided access to graph files (e.g. BED files) for the called peaks.

Data access links<br>*May remain private before publication.*	GSE284609

Methodology

Replicates

Single replicates of each antibody and genotype were conducted

Sequencing depth

All samples were sequenced using paired-end reads.

sample	total reads	trimmed reads	uniquely mapped reads	mapped spike-in
exp1_A498_sgCtrl_IgG	4080749	2664744	2259310	208975
exp1_A498_sgCtrl_KDM5C	8175882	7987089	7826948	96292
exp2_A498_sgCtrl_IgG	1216073	852921	774921	36909
exp2_A498_sgCtrl_H3K4me1	19548480	18995955	18748756	62449
exp2_A498_sgKDM5C_4_H3K4me1	8897681	7457898	7323676	18494
exp2_A498_sgKDM5C_5_H3K4me1	7561066	7039311	6941000	25203
exp2_A498_sgCtrl_H3K4me3	9525064	8958405	8824947	31584
exp2_A498_sgKDM5C_4_H3K4me3	8549869	7377853	7235498	32214
exp2_A498_sgKDM5C_5_H3K4me3	12615063	11559481	11352712	43620
exp2_A498_sgCtrl_H3K27me3	4183186	4079523	4031276	8792
exp2_A498_sgKDM5C_4_H3K27me3	13597865	13340087	13213455	27140
exp2_A498_sgKDM5C_5_H3K27me3	17804265	17580479	17427030	34081
exp2_A498_sgCtrl_H3K27ac	9763870	8762705	8277962	285227
exp2_A498_sgKDM5C_4_H3K27ac	10233550	9484042	8445064	465967
exp2_A498_sgKDM5C_5_H3K27ac	5743962	4370606	3542152	137204
exp3_786O_sgCtrl_IgG_for_Ab_H3K4me1	31889118	31518791	27184796	2066807
exp3_786O_sgCtrl_H3K4me1	20448076	20400015	18588436	382493
exp3_786O_sgKDM5D_2_H3K4me1	25651176	25574869	23494341	539303
exp3_786O_sgKDM5D_4_H3K4me1	30304031	30221109	27174833	1137041
exp3_786O_sgKDM5C_4_H3K4me1	19833465	18801732	16477804	854517
exp3_786O_sgKDM5C_5_H3K4me1	35352276	35137975	31891133	1052402
exp3_786O_sgKDM5C_4_sgKDM5D_2_H3K4me1	32784691	32673013	29809663	1017999
exp3_786O_sgKDM5C_4_sgKDM5D_4_H3K4me1	27229979	27145287	24676449	752111
exp3_786O_sgKDM5C_5_sgKDM5D_2_H3K4me1	31233168	31108708	28921787	909974
exp3_786O_sgKDM5C_5_sgKDM5D_4_H3K4me1	3409772	33812589	29712600	2148007
exp3_786O_sgCtrl_IgG_for_Ab_KDM5C_KDM5D	13530682	13483557	12499040	525694
exp3_786O_sgCtrl_KDM5C	10740557	10724058	10014336	248239
exp3_786O_sgKDM5D_2_KDM5C	6905503	6890878	6358487	218406
exp3_786O_sgKDM5D_4_KDM5C	8329494	8318649	7781933	247049
exp3_786O_sgKDM5C_4_KDM5C	12212142	12169624	11305516	391840
exp3_786O_sgKDM5C_5_KDM5C	7311976	7285141	6668151	200722
exp3_786O_sgCtrl_KDM5D	14076269	13984735	13065410	279563
exp3_786O_sgKDM5D_2_KDM5D	1222136	12179664	11575711	334062
exp3_786O_sgKDM5D_4_KDM5D	9606331	9556521	8703262	362472
exp3_786O_sgKDM5C_4_KDM5D	9075307	8981304	8259639	323016
exp3_786O_sgKDM5C_5_KDM5D	19300668	19216386	18134231	668236

Antibodies

One of the following antibodies were added to the appropriate reaction: IgG negative control (Cell Signaling Technology, #58802), anti-KDM5C (Abcam, ab34718), anti-KDM5D (BethylLab A301-751A), anti-H3K4me1 (Epicypher, 13-0040), anti-H3K4me3 (ActiveMotif, 39159), anti-H3K27ac (Epicypher, 13-0045), anti-H3K27me3 (Cell Signaling Technology, #9733).

Peak calling parameters

Raw FASTQ files were subjected to quality control and adapters and low-quality ends were trimmed using Trim Galore (version 0.6.6) with default settings. The alignment was performed using Bowtie2 (version 2.4.5) (35). The human reads were aligned to the UCSC hg19 reference genome and the spike-in control accordingly to the D. melanogaster UCSC dm6 reference genome for A498 experiments or E. coli NCBI K12 MG1655 reference genome for 786-O experiments. Based on the resulting BAM files, BigWig files were generated using bamCoverage from deepTools (version 3.5.0) (36) which can be used to visualize the coverage via the Integrative Genomics Viewer (IGV) (37). Additionally, for each sample, including the IgG control, the total number of aligned reads for both human and spike-in were calculated by samtools (version 1.16.1) flagstat (38) and the reads were normalized. First, the ratio of spike-in (spike) and human (genome) reads was calculated for each experimental sample (expr) (formula 1) and the IgG control (ctrl) (formula 2). Subsequently, a size factor was calculated by dividing the ratio of the control by the ratio of the experimental sample (formula 3). While the normalized reads for the experimental sample simply are the total human reads (exprgenome), the control is normalized for each experimental sample by multiplying the appropriate size factor with the total human reads from the IgG control (ctrlgenome) (formula 4) resulting in norm_ctrlgenome.

$$ratio\_expr = expr\_spike / expr\_genome$$

$$ratio\_ctrl = ctrl\_spike / ctrl\_genome$$

$$size\_factor = ratio\_ctrl / ratio\_expr$$

$$norm\_ctrl\_genome = size\_factor * ctrl\_genome$$

These normalized reads were used to generate tag directories using makeTagDirectory with the option -totalReads from Homer (Hypergeometric Optimization of Motif Enrichment) (version 4.11) (39) which parses through the alignment file and splits the tags into separate files based on the chromosomes. These tag directories were further used for computing coverage and peak calling.

For A498 cells treated with H3K27ac antibodies, only few peaks were detected based on the previously described normalization method. To this aim, another normalization method was used for the affected samples which resulted in a higher number of peaks for downstream analyses. For both the experimental and control samples, the mapped reads (samplegenome) were first normalized

ChIP-seq

Files in database submission

*processed data file processed data file *raw file raw file
exp1_A498_sgCtrl_IgG.bw exp1_A498_sgCtrl_IgG_R1.fastq.gz exp1_A498_sgCtrl_IgG_R2.fastq.gz
exp1_A498_sgCtrl_KDM5C.bw exp1_A498_sgCtrl_KDM5C.Clean.bed exp1_A498_sgCtrl_KDM5C_R1.fastq.gz
exp1_A498_sgCtrl_KDM5C_R2.fastq.gz
exp2_A498_sgCtrl_IgG.bw exp2_A498_sgCtrl_IgG_R1.fastq.gz exp2_A498_sgCtrl_IgG_R2.fastq.gz
exp2_A498_sgCtrl_H3K4me1.bw exp2_A498_sgCtrl_H3K4me1.Clean.bed exp2_A498_sgCtrl_H3K4me1_R1.fastq.gz
exp2_A498_sgCtrl_H3K4me1_R2.fastq.gz
exp2_A498_sgKDM5C_4_H3K4me1.bw exp2_A498_sgKDM5C_4_H3K4me1.Clean.bed
exp2_A498_sgKDM5C_4_H3K4me1_R1.fastq.gz exp2_A498_sgKDM5C_4_H3K4me1_R2.fastq.gz
exp2_A498_sgKDM5C_5_H3K4me1.bw exp2_A498_sgKDM5C_5_H3K4me1.Clean.bed
exp2_A498_sgKDM5C_5_H3K4me1_R1.fastq.gz exp2_A498_sgKDM5C_5_H3K4me1_R2.fastq.gz
exp2_A498_sgCtrl_H3K4me3.bw exp2_A498_sgCtrl_H3K4me3.Clean.bed exp2_A498_sgCtrl_H3K4me3_R1.fastq.gz
exp2_A498_sgCtrl_H3K4me3_R2.fastq.gz
exp2_A498_sgKDM5C_4_H3K4me3.bw exp2_A498_sgKDM5C_4_H3K4me3.Clean.bed
exp2_A498_sgKDM5C_4_H3K4me3_R1.fastq.gz exp2_A498_sgKDM5C_4_H3K4me3_R2.fastq.gz
exp2_A498_sgKDM5C_5_H3K4me3.bw exp2_A498_sgKDM5C_5_H3K4me3.Clean.bed
exp2_A498_sgKDM5C_5_H3K4me3_R1.fastq.gz exp2_A498_sgKDM5C_5_H3K4me3_R2.fastq.gz
exp2_A498_sgCtrl_H3K27me3.bw exp2_A498_sgCtrl_H3K27me3.Clean.bed exp2_A498_sgCtrl_H3K27me3_R1.fastq.gz
exp2_A498_sgCtrl_H3K27me3_R2.fastq.gz
exp2_A498_sgKDM5C_4_H3K27me3.bw exp2_A498_sgKDM5C_4_H3K27me3.Clean.bed
exp2_A498_sgKDM5C_4_H3K27me3_R1.fastq.gz exp2_A498_sgKDM5C_4_H3K27me3_R2.fastq.gz
exp2_A498_sgKDM5C_5_H3K27me3.bw exp2_A498_sgKDM5C_5_H3K27me3.Clean.bed
exp2_A498_sgKDM5C_5_H3K27me3_R1.fastq.gz exp2_A498_sgKDM5C_5_H3K27me3_R2.fastq.gz
exp2_A498_sgCtrl_H3K27ac.bw exp2_A498_sgCtrl_H3K27ac.Clean.bed exp2_A498_sgCtrl_H3K27ac_R1.fastq.gz
exp2_A498_sgCtrl_H3K27ac_R2.fastq.gz
exp2_A498_sgKDM5C_4_H3K27ac.bw exp2_A498_sgKDM5C_4_H3K27ac.Clean.bed
exp2_A498_sgKDM5C_4_H3K27ac_R1.fastq.gz exp2_A498_sgKDM5C_4_H3K27ac_R2.fastq.gz
exp2_A498_sgKDM5C_5_H3K27ac.bw exp2_A498_sgKDM5C_5_H3K27ac.Clean.bed
exp2_A498_sgKDM5C_5_H3K27ac_R1.fastq.gz exp2_A498_sgKDM5C_5_H3K27ac_R2.fastq.gz
exp3_786O_sgCtrl_IgG_for_Ab_H3K4me1.bw exp3_786O_sgCtrl_IgG_for_Ab_H3K4me1_R1.fastq.gz
exp3_786O_sgCtrl_IgG_for_Ab_H3K4me1_R2.fastq.gz
exp3_786O_sgCtrl_H3K4me1.bw exp3_786O_sgCtrl_H3K4me1.Clean.bed exp3_786O_sgCtrl_H3K4me1_R1.fastq.gz
exp3_786O_sgCtrl_H3K4me1_R2.fastq.gz
exp3_786O_sgKDM5D_2_H3K4me1.bw exp3_786O_sgKDM5D_2_H3K4me1.Clean.bed
exp3_786O_sgKDM5D_2_H3K4me1_R1.fastq.gz exp3_786O_sgKDM5D_2_H3K4me1_R2.fastq.gz
exp3_786O_sgKDM5D_4_H3K4me1.bw exp3_786O_sgKDM5D_4_H3K4me1.Clean.bed
exp3_786O_sgKDM5D_4_H3K4me1_R1.fastq.gz exp3_786O_sgKDM5D_4_H3K4me1_R2.fastq.gz
exp3_786O_sgKDM5C_4_H3K4me1.bw exp3_786O_sgKDM5C_4_H3K4me1.Clean.bed
exp3_786O_sgKDM5C_4_H3K4me1_R1.fastq.gz exp3_786O_sgKDM5C_4_H3K4me1_R2.fastq.gz
exp3_786O_sgKDM5C_5_H3K4me1.bw exp3_786O_sgKDM5C_5_H3K4me1.Clean.bed
exp3_786O_sgKDM5C_5_H3K4me1_R1.fastq.gz exp3_786O_sgKDM5C_5_H3K4me1_R2.fastq.gz
exp3_786O_sgKDM5C_4_sgKDM5D_2_H3K4me1.bw exp3_786O_sgKDM5C_4_sgKDM5D_2_H3K4me1.Clean.bed
exp3_786O_sgKDM5C_4_sgKDM5D_2_H3K4me1_R1.fastq.gz exp3_786O_sgKDM5C_4_sgKDM5D_2_H3K4me1_R2.fastq.gz
exp3_786O_sgKDM5C_4_sgKDM5D_4_H3K4me1.bw exp3_786O_sgKDM5C_4_sgKDM5D_4_H3K4me1.Clean.bed
exp3_786O_sgKDM5C_4_sgKDM5D_4_H3K4me1_R1.fastq.gz exp3_786O_sgKDM5C_4_sgKDM5D_4_H3K4me1_R2.fastq.gz
exp3_786O_sgKDM5C_5_sgKDM5D_2_H3K4me1.bw exp3_786O_sgKDM5C_5_sgKDM5D_2_H3K4me1.Clean.bed
exp3_786O_sgKDM5C_5_sgKDM5D_2_H3K4me1_R1.fastq.gz exp3_786O_sgKDM5C_5_sgKDM5D_2_H3K4me1_R2.fastq.gz
exp3_786O_sgKDM5C_5_sgKDM5D_4_H3K4me1.bw exp3_786O_sgKDM5C_5_sgKDM5D_4_H3K4me1.Clean.bed
exp3_786O_sgKDM5C_5_sgKDM5D_4_H3K4me1_R1.fastq.gz exp3_786O_sgKDM5C_5_sgKDM5D_4_H3K4me1_R2.fastq.gz
exp3_786O_sgCtrl_IgG_for_Ab_KDM5C_KDM5D.bw exp3_786O_sgCtrl_IgG_for_Ab_KDM5C_KDM5D_R1.fastq.gz
exp3_786O_sgCtrl_IgG_for_Ab_KDM5C_KDM5D_R2.fastq.gz
exp3_786O_sgCtrl_KDM5C.bw exp3_786O_sgCtrl_KDM5C.Clean.bed exp3_786O_sgCtrl_KDM5C_R1.fastq.gz
exp3_786O_sgCtrl_KDM5C_R2.fastq.gz
exp3_786O_sgKDM5D_2_KDM5C.bw exp3_786O_sgKDM5D_2_KDM5C.Clean.bed
exp3_786O_sgKDM5D_2_KDM5C_R1.fastq.gz exp3_786O_sgKDM5D_2_KDM5C_R2.fastq.gz
exp3_786O_sgKDM5D_4_KDM5C.bw exp3_786O_sgKDM5D_4_KDM5C.Clean.bed
exp3_786O_sgKDM5D_4_KDM5C_R1.fastq.gz exp3_786O_sgKDM5D_4_KDM5C_R2.fastq.gz
exp3_786O_sgKDM5C_4_KDM5C.bw exp3_786O_sgKDM5C_4_KDM5C.Clean.bed
exp3_786O_sgKDM5C_4_KDM5C_R1.fastq.gz exp3_786O_sgKDM5C_4_KDM5C_R2.fastq.gz
exp3_786O_sgKDM5C_5_KDM5C.bw exp3_786O_sgKDM5C_5_KDM5C.Clean.bed
exp3_786O_sgKDM5C_5_KDM5C_R1.fastq.gz exp3_786O_sgKDM5C_5_KDM5C_R2.fastq.gz
exp3_786O_sgCtrl_KDM5D.bw exp3_786O_sgCtrl_KDM5D.Clean.bed exp3_786O_sgCtrl_KDM5D_R1.fastq.gz
exp3_786O_sgCtrl_KDM5D_R2.fastq.gz
exp3_786O_sgKDM5D_2_KDM5D.bw exp3_786O_sgKDM5D_2_KDM5D.Clean.bed
exp3_786O_sgKDM5D_2_KDM5D_R1.fastq.gz exp3_786O_sgKDM5D_2_KDM5D_R2.fastq.gz
exp3_786O_sgKDM5D_4_KDM5D.bw exp3_786O_sgKDM5D_4_KDM5D.Clean.bed
exp3_786O_sgKDM5D_4_KDM5D_R1.fastq.gz exp3_786O_sgKDM5D_4_KDM5D_R2.fastq.gz
exp3_786O_sgKDM5C_4_KDM5D.bw exp3_786O_sgKDM5C_4_KDM5D.Clean.bed
exp3_786O_sgKDM5C_4_KDM5D_R1.fastq.gz exp3_786O_sgKDM5C_4_KDM5D_R2.fastq.gz
exp3_786O_sgKDM5C_5_KDM5D.bw exp3_786O_sgKDM5C_5_KDM5D.Clean.bed
exp3_786O_sgKDM5C_5_KDM5D_R1.fastq.gz exp3_786O_sgKDM5C_5_KDM5D_R2.fastq.gz

Genome browser session
(e.g. UCSC)

No longer applicable

to the number of initially anticipated reads per sample (formula 5), as well as to the sum of reads for all samples (formula 6), resulting in formula 7. The ratio of mapped reads to the genome (formula 8), as well as to the spike-in (formula 9) was calculated by dividing each sample by the control. Finally, formula 10 resulted in the normalized reads for each sample used to generate tag directories.

sample_totalReads= (sample_aimedReads)/(sample_totalReadsProcessed)

all_totalReads= (all_aimedReads)/(all_totalReadsProcessed)

norm_totalReads=sample_genome* (sample_totalReads)/(all_totalReads)

ratio_genome= (sample_genome)/(ctrl_genome)

ratio_spike= (sample_spike)/(ctrl_spike)

norm_sample_genome= (norm_totalReads)/((ratio_genome)/(ratio_spike))

CUT&RUN peak calling
Peaks were called using Homer's findPeaks function using the histone mode with default parameters. The option -C was set to 0 to disable filtering based on clonal signals, since a high frequency of fragments with identical ends can generally arise from different cells in CUT&RUNseq experiments. Experimental samples were directly compared to the IgG background control and only peaks with 4-fold more tags in the experimental sample compared to the control (sequencing-depth independent), as well as with a cumulative Poisson p-value ≤ 0.0001 (sequencing depth dependent) were kept. Peaks within blacklisted regions from ENCODE (40), such as repetitive sequences, were excluded using intersectBed from BEDTools (version 2.27.1) (41). The peak calling analysis results in a bed file with all identified peaks with their genomic location and several statistics.

In order to explore the peaks, unique and overlapping peaks were identified between the different samples using Homer mergePeaks with the distance option -d given. This option requires the peaks to have a specific overlap between the start and end coordinates in order to be merged. For visualizations of the signal intensity around peaks or other genomic locations, heatmaps were generated using functions from deepTools. First, bamCompare was used to compare the read counts of each experimental sample to the IgG control with the option --scaleFactors 1:S, whereas S is equal to ratioexpr (formula 1 above) divided by ratioctrl (formula 2 above). The resulting signal intensities are represented as the log2 of the ratio of the sequencing depth normalized reads. Next, computeMatrix was used to generate a matrix of signal intensities up- and downstream of the peak centers which was subjected to plotHeatmap and plotProfile for visualizations.

Data quality

See above

Software

See above
