# Supplementary material for: KDM5C and KDM5D mutations have different consequences in clear cell renal cell carcinoma cells
Source: Commun Biol. 2025 Feb 15;8:244. doi: 10.1038/s42003-025-07695-8 (PMC11830100; doi:10.1038/s42003-025-07695-8)
Supplement: Supplementary file 8 — Peer Review [file 42003_2025_7695_MOESM8_ESM.pdf]

nature portfolio

Peer Review File

KDM5C and KDM5D mutations have different consequences in clear cell renal cell carcinoma cells

Corresponding Author: Professor Ian Frew

This manuscript has been previously reviewed at another journal. This document only contains information relating to versions considered at Communications Biology.

This file contains all reviewer reports in order by version, followed by all author rebuttals in order by version.

Version 0:

Reviewer comments:

Reviewer #1

(Remarks to the Author)
The manuscript “KDM5C and KDM5D mutations have different and opposing consequences in ccRCC cells” address the role of epigenetic regulation in the carcinogenesis of ccRCC. Specifically, authors focused on the role of histone demethylases, and have explored hypotheses suggesting that either KDM5C mutation, loss of part or all of the X chromosome or loss of part or all of the Y chromosome causing loss of KDM5D might act to promote ccRCC formation. Authors performed a series of in vitro and in vivo experiments examining the effects of mutation of KDM5C and/or KDM5D in human ccRCC cell lines. To mimic the effects of loss of function mutations in KDM5C in the background of other ccRCC-specific mutations authors employed CRISPR-Cas9 mutagenesis to compare the effects of KDM5C mutation across multiple ccRCC cell lines.

Authors showed that KDM5C binding was associated with different patterns of histone modifications at promoters, enhancers, genes and intergenic regions of the genome. Across all KDM5C binding sites, levels of H3K4me1 were reduced in the KDM5C mutant cells, consistent with KDM5C functioning as a major H3K4 demethylase.
Interestingly, only 11% of the upregulated genes and 5% of the downregulated genes were associated with KDM5C-bound promoters. These observations suggested that the predominant mode of KDM5C-mediated control of gene expression is not via direct binding and regulation of H3K4 methylation at promoters. Authors suggested that the majority of the observed gene expression changes are due to secondary effects of KDM5C mutation rather than being direct target genes.
This study provide novel information on KDM5C function, suggesting that KDM5C affects the underlying transcriptional state of the ccRCC cells. Loss of KDM5C function alters gene expression in each individual tumor in combination with other genetic mutations and signaling pathway states that govern the overall transcriptional output in that tumor.
There few minor points to address:
1) Fig. 1 (B) KDM5C western blot of ccRCC cell lines show different levels of KDM5C protein in examined cells line. In addition to mutations, which mechanisms can be involved in regulation of KDM5C protein expression?
2) Fig. 1 (F) Xenograft assays of the CAKI-2 cells indicated almost 2 weeks delay in tumor formation in KDM5C mutant cells as compared to control cells. Please provide explanation for these observations.
3) This study demonstrated that KDM5C mutation slightly reduced metastatic propensity, while KDM5D mutation completely blocked xenograft growth. KDM5C co-mutation in KDM5D mutant cells completely restored tumor growth and metastasis. Please discuss these findings in the context of treatment with KDM5 inhibitors.
4) Authors demonstrated that KDM5C mutation allows KDM5D mutant cells to form tumors. This genetic relationship between KDM5C and KDM5D mutation is consistent with the human data that indicates that KDM5C mutant male ccRCC tumors display concomitant loss of KDM5D function through Y chromosome loss. Please discuss these observations in a broader context, highlighting the sex-specific differences in cancer biology.

Reviewer #2

(Remarks to the Author)
Muller et al. analyzed the fonctional impact of KDM5C in ccRCC tumours through the introduction of KDM5C mutation into two male and two female KDM5C wild type ccRCC cell lines. They furthermore showed that KDM5C and KDM5D mutations

have different and opposing consequences in ccRCC cells and they then demonstrated that that gene expression changes induced by KDM5C or KDM5D mutation are apparently unrelated to the direct functions of these proteins at the relevant gene promoters or enhancers. The authors used useful methodologies including CRISPR/CAs9 loss of function, cut-and-run and RNAseq in several cell lines that are relevant to the disease. relevance has been shown by using patients WES data. Overall, the manuscript is very well written and the conclusions are original. The manuscript would be a great addition to the field. I would love to see it published.

Minor comments:

1) authors need to comment extensively on the differences of the results obtained in their experiments as compared to Outchkourov (ref 22 in the manuscript)

2) Authors need to comment on upregulation of interferon response in KDM5D mutant 786-O cells. How relevance their model is to understand immune evasion and what further experiments might be needed in the future to clarify this?

3) It would be important that the authors add a section on the discussion to highlight other possible functions of KDM5C/D proteins beside their direct functions at the relevant gene promoters or enhancers identified through RNAseq and cut and run.

Version 1:

Reviewer comments:

Reviewer #1

(Remarks to the Author)
Authors revised manuscript and addressed comments.

Reviewer #2

(Remarks to the Author)
thanks for answering all the questions.

Open Access This Peer Review File is licensed under a Creative Commons Attribution 4.0 International License, which permits use, sharing, adaptation, distribution and reproduction in any medium or format, as long as you give appropriate credit to the original author(s) and the source, provide a link to the Creative Commons license, and indicate if changes were made.
In cases where reviewers are anonymous, credit should be given to 'Anonymous Referee' and the source.
The images or other third party material in this Peer Review File are included in the article’s Creative Commons license, unless indicated otherwise in a credit line to the material. If material is not included in the article’s Creative Commons license and your intended use is not permitted by statutory regulation or exceeds the permitted use, you will need to obtain permission directly from the copyright holder.

To view a copy of this license, visit https://creativecommons.org/licenses/by/4.0/

Reviewers' comments:

Reviewer #1 (Remarks to the Author):

The manuscript "KDM5C and KDM5D mutations have different and opposing consequences in ccRCC cells" address the role of epigenetic regulation in the carcinogenesis of ccRCC. Specifically, authors focused on the role of histone demethylases, and have explored hypotheses suggesting that either KDM5C mutation, loss of part or all of the X chromosome or loss of part or all of the Y chromosome causing loss of KDM5D might act to promote ccRCC formation. Authors performed a series of in vitro and in vivo experiments examining the effects of mutation of KDM5C and/or KDM5D in human ccRCC cell lines. To mimic the effects of loss of function mutations in KDM5C in the background of other ccRCC-specific mutations authors employed CRISPR-Cas9 mutagenesis to compare the effects of KDM5C mutation across multiple ccRCC cell lines.

Authors showed that KDM5C binding was associated with different patterns of histone modifications at promoters, enhancers, genes and intergenic regions of the genome. Across all KDM5C binding sites, levels of H3K4me1 were reduced in the KDM5C mutant cells, consistent with KDM5C functioning as a major H3K4 demethylase.
Interestingly, only 11% of the upregulated genes and 5% of the downregulated genes were associated with KDM5C-bound promoters. These observations suggested that the predominant mode of KDM5C-mediated control of gene expression is not via direct binding and regulation of H3K4 methylation at promoters. Authors suggested that the majority of the observed gene expression changes are due to secondary effects of KDM5C mutation rather than being direct target genes.
This study provide novel information on KDM5C function, suggesting that KDM5C affects the underlying transcriptional state of the ccRCC cells. Loss of KDM5C function alters gene expression in each individual tumor in combination with other genetic mutations and signaling pathway states that govern the overall transcriptional output in that tumor.

We are most grateful to the reviewer for the investment of their time in reviewing our work, for the positive endorsement of our study and for the helpful suggestions.

There few minor points to address:
1) Fig. 1 (B) KDM5C western blot of ccRCC cell lines show different levels of KDM5C protein in examined cells line. In addition to mutations, which mechanisms can be involved in regulation of KDM5C protein expression?
Thank you for this suggestion. We are reluctant to overinterpret differences between different cell lines as these experiments were not designed to quantitively compare protein abundance of KDM5C between different cancer cell lines, which can differ from one another in so many ways (for example differences in expression levels of the "loading control" VINCULIN between cell lines could rather account for the apparent relative difference in KDM5C protein abundance). This experiment was designed to show that KDM5C is expressed in all of the cell lines to allow us to develop the isogenic CRISPR-Cas9 WT and *KDM5C* mutant sets of cell lines which were directly compared in the controlled genetic setting within each cancer cell line. Nonetheless, we have now altered the description of this western blot to directly address this comment as follows (additions/alterations in blue text)…..

KDM5C mRNA was detected at similar levels in 786-O, CAKI-2, A498 and 769-P cells (Supplementary Figure 2) and western blotting confirmed the expression of KDM5C in all four cell lines with *KDM5C* mutant RCC4 cells serving as a negative control for the antibody (Figure 1B). Variations in KDM5C protein abundance relative to the VINCULIN loading control did not correlate with sex of the cell line with male 786-O and female A498 cells expressing similar levels, male CAKI-2 cells expressing lower levels and female 769-P cells expressing the highest.

2) Fig. 1 (F) Xenograft assays of the CAKI-2 cells indicated almost 2 weeks delay in tumor formation in KDM5C mutant cells as compared to control cells. Please provide explanation for these observations.

Thank you for this comment. We have included the following sentences in the Results section.

In CAKI-2 cells, the enrichment of the GO term “Negative regulation of cell cycle processes” and downregulation of genes associated with GO, Hallmark and KEGG terms “Oxidative phosphorylation” are potentially consistent with the delay in xenograft growth induced by *KDM5C* mutation in this cell line.

3) This study demonstrated that KDM5C mutation slightly reduced metastatic propensity, while KDM5D mutation completely blocked xenograft growth. KDM5C co-mutation in KDM5D mutant cells completely restored tumor growth and metastasis. Please discuss these findings in the context of treatment with KDM5 inhibitors.
While we appreciate that it is always valuable to consider the translational perspective and possibility of pharmacological targeting of key cellular enzymes, we do not believe that a discussion of KDM5 inhibitors in this particular context is warranted. While theoretically our data argues that specific inhibition of KDM5D might mimic the effect of genetic mutation of *KDM5D* which blocked xenograft tumour growth, to the best of our knowledge there are no specific KDM5D inhibitors that have been developed. General inhibition of KDM5 enzymes, which would also lead to inhibition of the tumour suppressor functions of KDM5C does not appear to be a good concept for ccRCC therapy.

4) Authors demonstrated that KDM5C mutation allows KDM5D mutant cells to form tumors. This genetic relationship between KDM5C and KDM5D mutation is consistent with the human data that indicates that KDM5C mutant male ccRCC tumors display concomitant loss of KDM5D function through Y chromosome loss. Please discuss these observations in a broader context, highlighting the sex-specific differences in cancer biology.
We agree that these are important points and had already discussed these issues in several sections of the Discussion. To further emphasise this point we have modified the final section of the discussion to more specifically highlight testable hypotheses that emerge from our work.

Finally, this study highlights that there may be fundamental genetic differences between ccRCCs in men and in women and contributes to the growing understanding that Y chromosome loss is not just a bystander event during the formation of many different tumour types in men, but rather contributes in different ways to the evolution and pathology of these diseases, as well as to sex-specific differences in tumour incidence and biology (17,32,33). For example, in bladder cancer, Y chromosome loss contributes to tumour immune evasion (23) while in colorectal carcinoma, upregulation of *KDM5D* expression alters cell adhesion, antigen presentation and anti-tumour immunity, accounting for differences between male and female tumours (34). These studies implicate Y chromosome status in crosstalk between tumour cells and immune cells. In this context it is notable that several genes involved in the interferon response are upregulated in *KDM5D* mutant 786-O cells, potentially reflecting activation of the STING-mediated immune response. STING activation induces the expression of several cytokines which can lead to the recruitment of different types of immune cells. Notably, *KDM5C* co-mutation abrogates the upregulation of the interferon responsive genes in *KDM5D* mutant cells. Given that advanced ccRCC tumours are treated with immune checkpoint therapies, it will be important to further characterise the consequences of loss of the Y chromosome and mutation of *KDM5C* in the context of male ccRCC tumour evolution and tumour cell interactions with the immune system to determine if these different types of genetic alterations may shape the tumour immune microenvironment. In addition to analyses of human ccRCC tumours segregated based on Y chromosome and *KDM5C* mutation status, functional experiments introducing *Kdm5c* and *Kdm5d* mutations into mouse autochthonous ccRCC models could be informative.

Reviewer #2 (Remarks to the Author):

Muller et al. analyzed the fonctional impact of KDM5C in ccRCC tumours through the introduction of KDM5C mutation into two male and two female KDM5C wild type ccRCC cell lines. They furthermore showed that KDM5C and KDM5D mutations have different and opposing consequences in ccRCC cells and they then demonstrated that that gene expression changes induced by KDM5C or KDM5D mutation are apparently unrelated to the direct functions of these proteins at the relevant gene promoters or enhancers. The authors used useful methodologies including CRISPR/CAs9 loss of function, cut-and-run and RNAseq in several cell lines that are relevant to the disease. relevance has been shown by using patients WES data. Overall, the manuscript is very well written and the conclusions are original. The manuscript would be a great addition to the field. I would love to see it published.

We are most grateful to the reviewer for the investment of their time in reviewing our work, for the positive endorsement of our study and for the helpful suggestions.

Minor comments:

1) authors need to comment extensively on the differences of the results obtained in their experiments as compared to Outchkourov (ref 22 in the manuscript)
Thank you for this suggestion. We have now added several new sections to the first part of the Discussion to directly address this comment as follows (additions/alterations in blue text)…..

"The *KDM5C* gene is mutated in approximately 20% of ccRCC metastases but the molecular mechanisms through which loss of KDM5C function contribute to tumour evolution and metastatic spread remain incompletely characterised. While KDM5C functions as an H3K4 demethylase and is proposed to thereby regulate chromatin states and associated gene transcription, our findings argue that regulation of a specific and common gene expression pattern is very unlikely to be the mechanism through which KDM5C acts as a ccRCC tumour suppressor protein. *KDM5C* mutation in ccRCC cell lines altered the expression of only 100-200 genes and there were essentially no overlaps in the differentially expressed genes across four different ccRCC cell lines. This argument however does not imply that transcriptional alterations induced by *KDM5C* mutation do not contribute to ccRCC. It is possible that loss of KDM5C function alters gene expression in each individual tumour in combination with the constellation of genetic mutations and signalling pathway states that govern the overall transcriptional output in that tumour. This model is in keeping with KDM5C's function as an enzyme that can demethylate H3K4 at many sites in the genome and thereby serve as an accessory enzyme to modulate gene expression driven by other factors, rather than itself being a direct driver of transcription. Consistent with this idea, a previous study using ES cells showed that in general, loss of KDM5C function alters the balance of H3K4me3 and H3K4me1 at promoters and enhancers to control the expression of some genes (22). However, it should be noted that this effect was not black and white at the individual gene level. KDM5C bound to several thousand sites in the genome but the expression of only a few hundred genes were altered upon *KDM5C* knockdown. Genes nearby KDM5C binding sites could either be upregulated or downregulated upon *KDM5C* knockdown, even when segregated based on levels of H3K4me3 at those sites."

2) Authors need to comment on upregulation of interferon response in KDM5D mutant 786-O cells. How relevance their model is to understand immune evasion and what further experiments might be needed in the future to clarify this?
Thank you for this suggestion. We have altered the last paragraph of the Discussion to address this issue as follows (additions/alterations in blue text)…..

"These studies implicate Y chromosome status in crosstalk between tumour cells and immune cells. In this context it is notable that several genes involved in the interferon response are upregulated in *KDM5D* mutant 786-O cells, potentially reflecting activation of the STING-mediated immune response. STING activation induces the expression of several cytokines which can lead to the recruitment of different types of immune cells. Notably, *KDM5C* co-mutation abrogates the upregulation of the interferon responsive genes in *KDM5D* mutant cells. Given that advanced ccRCC tumours are treated

with immune checkpoint therapies, it will be important to further characterise the consequences of loss of the Y chromosome and mutation of *KDM5C* in the context of ccRCC tumour evolution and tumour cell interactions with the immune system to determine if these different types of genetic alterations may shape the tumour immune microenvironment. In addition to analyses of human ccRCC tumours segregated based on Y chromosome and *KDM5C* mutation status, functional experiments introducing *Kdm5c* and *Kdm5d* mutations into mouse autochthonous ccRCC models could be informative."

3) It would be important that the authors add a section on the discussion to highlight other possible functions of KDM5C/D proteins beside their direct functions at the relevant gene promoters or enhancers identified through RNAseq and cut and run.
Thank you for this suggestion. We have now added several new sections to the first part of the Discussion to directly address this comment as follows (additions/alterations in blue text)…..

"In A498 and 786-O cells we were unable to detect KDM5C binding to promoters and enhancers of the majority of genes whose expression is altered upon *KDM5C* mutation, suggesting that KDM5C might also impact gene expression through activities outside of promoters and enhancers, such as in gene bodies or in intergenic regions where KDM5C binding in A498 cells also correlates with enrichment of the H3K4me1 mark, which is produced as a result of KDM5 enzymatic activity. These regions may potentially also contain transcriptional regulatory elements that are controlled by the balance of H3K4 tri- and mono-methylation. It also cannot be excluded that secondary effects of *KDM5C* mutation dominate the overall contribution to alteration of the transcriptome. The absence of histone demethylase activity of KDM5C in *KDM5C* mutant cells may also contribute to tumour evolution by inducing DNA replication stress (24) or genomic instability (25). Biochemical studies have also suggested that KDM5C and KDM5D might have numerous additional non-histone substrates that potentially extend the functions of KDM5 family enzymes beyond regulation of histones and transcription (26). While these putative substrates remain to be validated and functionally characterised, it is plausible that some of these proteins may also be relevant to ccRCC biology in *KDM5C* mutant and/or Y-chromosome loss tumours.
